# Absent in melanoma 2 mediates aging‐related cognitive dysfunction by acting on complement‐dependent microglial phagocytosis

**DOI:** 10.1111/acel.13860

**Published:** 2023-05-12

**Authors:** Lei Ye, Shu Shu, Junqiu Jia, Min Sun, Siyi Xu, Xinyu Bao, Huijie Bian, Yi Liu, Meijuan Zhang, Xiaolei Zhu, Feng Bai, Yun Xu

**Affiliations:** ^1^ Department of Neurology, Nanjing Drum Tower Hospital, Medical School and The State Key Laboratory of Pharmaceutical Biotechnology, Institute of Translational Medicine for Brain Critical Diseases, Medical School Nanjing University Nanjing China; ^2^ Jiangsu Key Laboratory for Molecular Medicine Medical School of Nanjing University Nanjing China; ^3^ Jiangsu Provincial Key Discipline of Neurology Nanjing China; ^4^ Nanjing Neurology Medical Center Nanjing China; ^5^ Nanjing Neuropsychiatry Clinic Medical Center Nanjing China

**Keywords:** aging, AIM2, complement, microglia, pattern separation, synaptic loss

## Abstract

Pattern separation (PS) dysfunction is a type of cognitive impairment that presents early during the aging process, and this deficit has been attributed to structural and functional alterations in the dentate gyrus (DG) of the hippocampus. Absent in melanoma 2 (AIM2) is an essential component of the inflammasome. However, whether AIM2 plays a role in aging‐associated cognitive dysfunction remains unclear. Here, we found that PS function was impaired in aging mice and was accompanied by marked synaptic loss and increased expression of AIM2 in the DG. Subsequently, we used an AIM2 overexpression virus and mice with AIM2 deletion to investigate the role of AIM2 in regulating PS function and synaptic plasticity and the mechanisms involved. Our study revealed that AIM2 regulates microglial activation during synaptic pruning in the DG region via the complement pathway, leading to impaired synaptic plasticity and PS function in aging mice. These results suggest a critical role for AIM2 in regulating synaptic plasticity and PS function and provide a new direction for ameliorating aging‐associated cognitive dysfunction.

## Graphical abstract

Pattern separation (PS) dysfunction is a cognitive impairment that presents early during the aging process, and this deficit has been attributed to structural and functional alterations in the dentate gyrus (DG) region of the hippocampus. Increased expression of absent in melanoma 2 in the DG region regulates microglial activation in synaptic pruning via the complement pathway, thereby leading to impaired synaptic plasticity and PS behavior in aging mice.

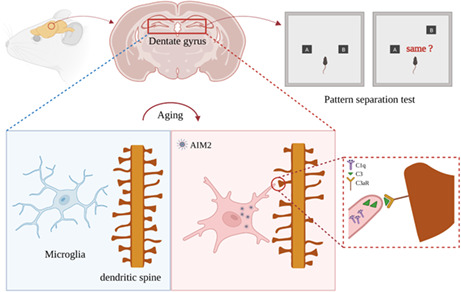

**Abbreviations**
AIM2: Absent in melanoma 2
AD: Alzheimer's disease
CNS: central nervous system
DG: dentate gyrus
LTP: long‐term potentiation
mEPSCs: Miniature excitatory postsynaptic currents
OPS: object pattern separation
PFA: paraformaldehyde
PS: Pattern separation
PBS: phosphate‐buffered saline
WT: wild‐type

## 1 INTRODUCTION

The aging of the population is becoming an increasingly serious global problem and has led to a significant increase in the incidence of dementia (Leung et al., 2022). There is an urgent need to find the specific mechanism of aging‐related cognitive impairment and prevent or slow its occurrence and development (Yang et al., 2021). Pattern separation (PS), which refers to the process of forming distinct memories from highly similar but slightly different events or stimuli, has been determined to be one of the early cognitive functions affected by aging, mild cognitive impairment and Alzheimer's disease (AD; Lee et al., 2020). Moreover, the dentate gyrus (DG) region in the hippocampus has been considered to play an important role in PS using various experimental approaches, from lesions to genetic manipulation in animal studies (Kita et al., 2021; Morris et al., 2012; van Dijk & Fenton, 2018).

Absent in melanoma 2 (AIM2), an essential component of the inflammasome, acts as a sensor of double‐stranded DNA and plays an important role in antiviral and antibacterial defenses, as well as in autoimmune diseases involving its own DNA (Broz & Dixit, 2016). The AIM2 inflammasome is significantly activated during neurodevelopment, and AIM2 knockout results in the accumulation of DNA damage in the central nervous system (CNS) and abnormal anxiety‐like behavior in mice (Lammert et al., 2020). In addition, AIM2 is mainly expressed in microglia, astrocytes, neurons and endothelial cells in the brain (Li et al., 2021; Xu et al., 2021). Our previous research showed that AIM2 protein levels were significantly upregulated in the hippocampus of AD mice, and AIM2 knockout mice showed increased neuronal dendrite branching, synaptic plasticity, and spatial learning and memory (Chen et al., 2019). However, the role of AIM2 in aging‐related cognitive dysfunction remains unclear.

Synaptic loss is a major pathological event in aging‐associated cognitive decline and neurodegenerative diseases (Hong et al., 2016; Pannese, 2011). It is generally known that microglia play a key role in regulating synaptic remodeling in the CNS. Abnormal microglial phagocytosis is involved in regulating synaptic pruning in aging and neurodegenerative diseases (Hong et al., 2016). A recent study showed that deletion of the microglial protein SIRPα results in increased synaptic loss and enhanced cognitive impairment mediated by microglial phagocytosis (Ding et al., 2021). In addition, C9orf72‐deficient microglia trigger age‐dependent increases in synaptic pruning of neurons, leading to learning and memory deficits in mice (Lall et al., 2021). Moreover, activation of the classical complement pathway promotes microglial phagocytosis‐mediated synaptic pruning (Hong et al., 2016).

In this study, we investigated the role of AIM2 in regulating PS behavioral impairment in aging and further explored the mechanisms by which AIM2 regulates synaptic loss in the hippocampal DG region. Our study demonstrated that AIM2 regulates microglial synaptic pruning of neurons in the DG region via the complement pathway, thereby affecting PS behavior in aging mice. These results reveal a novel role of AIM2 in the regulation of neural synaptic function and provide a new direction for improving aging‐associated cognitive dysfunction.

## 2 RESULTS

### 2.1 Aging mice displayed impaired PS behavior associated with elevated AIM2 expression in the DG region

**FIGURE 1:**
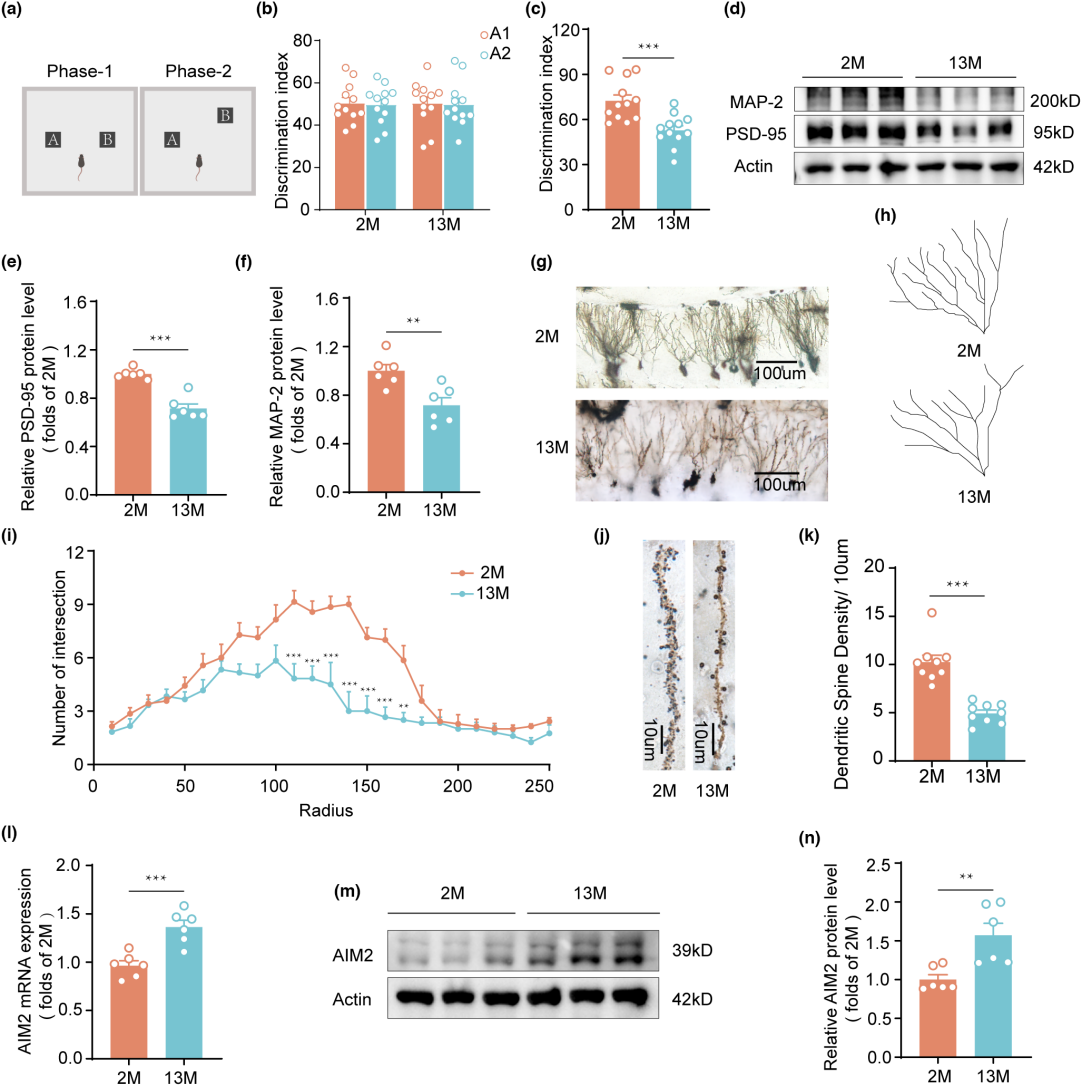
Aging mice displayed impaired PS behavior associated with elevated AIM2 expression in the DG region. (a) Schematic diagram of the OPS test. The time spent exploring two identical objects during the training phase (b) and the displaced object during the test session (c) by 2‐ and 13‐month‐old male mice in the OPS test was recorded. *n* = 12 for each group. *t*(22) = 4, *p* = 0.006. (d) PSD‐95 and MAP‐2 protein expression levels in the DG of 2‐ and 13‐month‐old male mice were analyzed using immunoblot assays. Quantification of the protein expression levels of PSD‐95 (e) and MAP‐2 (f) normalized to β‐actin as a loading control. *n* = 6 for each group. *t*(10) = 6.848, *p* < 0.0001 for PSD‐95; *t*(10) = 3.485, *p* = 0.0059 for MAP‐2. (g) Low‐magnification view of the DG region delineated by Golgi staining. (h) Representative traces of DG neurons. (i) The numbers of intersections at different distances from the soma in 2‐month‐old (*n* = 2 neurons/mouse, *N* = 3 mice/group) and 13‐month‐old (*n* = 2–3 neurons/mouse, *N* = 3 mice/group) male mice were counted. *F*(1,11) = 35.19, *p* < 0.0001. (j) Representative images of dendritic spines in DG neurons in 2‐ and 13‐month‐old male mice. (k) Quantitative analysis of the dendritic spine density in 2‐month‐old (*n* = 3 spines/mouse, *N* = 3 mice/group) and 13‐month‐old (*n* = 3 spines/mouse, *N* = 3 mice/group) male mice. *t*(16) = 6.651, *p* < 0.0001. (l) The mRNA level of AIM2 was measured in 2‐month‐old (*n* = 6 mice) and 13‐month‐old (*n* = 6 mice) male mice, and the expression of GAPDH mRNA was used for normalization. *t*(10) = 4.622, *p* = 0.0009. (m, n) The level of AIM2 in the DG of 2‐ and 13‐month‐old male mice was determined by western blotting, and the intensities of the protein bands normalized to those of the β‐actin band are shown. *n* = 6 for each group. *t*(10) = 3.437, *p* = 0.0064. Data were shown as mean ± SEM. Unpaired two‐tailed *t*‐test for c, e, f, k, l and n. Two‐way ANOVA followed by Bonferroni's post hoc test for i. **p < 0.01, ***p < 0.001.

First, we examined motor and cognitive function in young (2 months) and aging (13 months) mice. In the Morris water maze and open field tests, the results revealed that 13‐month‐old mice did not display significant differences in spatial learning and memory, locomotor activity or anxiety‐related behaviors compared to 2‐month‐old mice (Figure S1). In the object pattern separation (OPS) task (Figure 1a), 13‐month‐old mice spent less time exploring the object in a new location, exhibiting significantly impaired performance relative to that of 2‐month‐old mice (Figure 1b,c).

Given the links between synaptic plasticity in the DG and PS (Jungenitz et al., 2018; Kita et al., 2021), we quantitatively evaluated the levels of synaptic markers in the DG of 2‐ and 13‐month‐old mice. The protein levels of the postsynaptic marker PSD‐95 and the neuritic marker MAP‐2 were significantly decreased in 13‐month‐old mice compared with 2‐month‐old mice (Figure 1d–f), but there was no significant difference in the expression of the presynaptic marker synapsin‐1 (SYN‐1; Figure S2). Consistent with these results, using Golgi staining, we found that neurons in the DG of 13‐month‐old mice had less dendritic complexity (Figure 1g–i) and spine density (Figure 1j,k) of neurons in the DG was reduced in 13‐month‐old mice compared with 2‐month‐old mice. In summary, these results revealed PS dysfunction and synaptic impairment in aging mice.

Our previous study found that AIM2 could activate microglia‐related inflammation in experimental stroke (Zhang et al., 2020). However, neuroinflammation is an important factor in aging‐related cognitive decline. Interestingly, our results indicated that the AIM2 levels in DG of 13‐month‐old mice were clearly elevated (Figure 1l–n).

### 2.2 AIM2 ^−/−^ mice displayed improved cognitive function and ameliorated synapse loss

**FIGURE 2:**
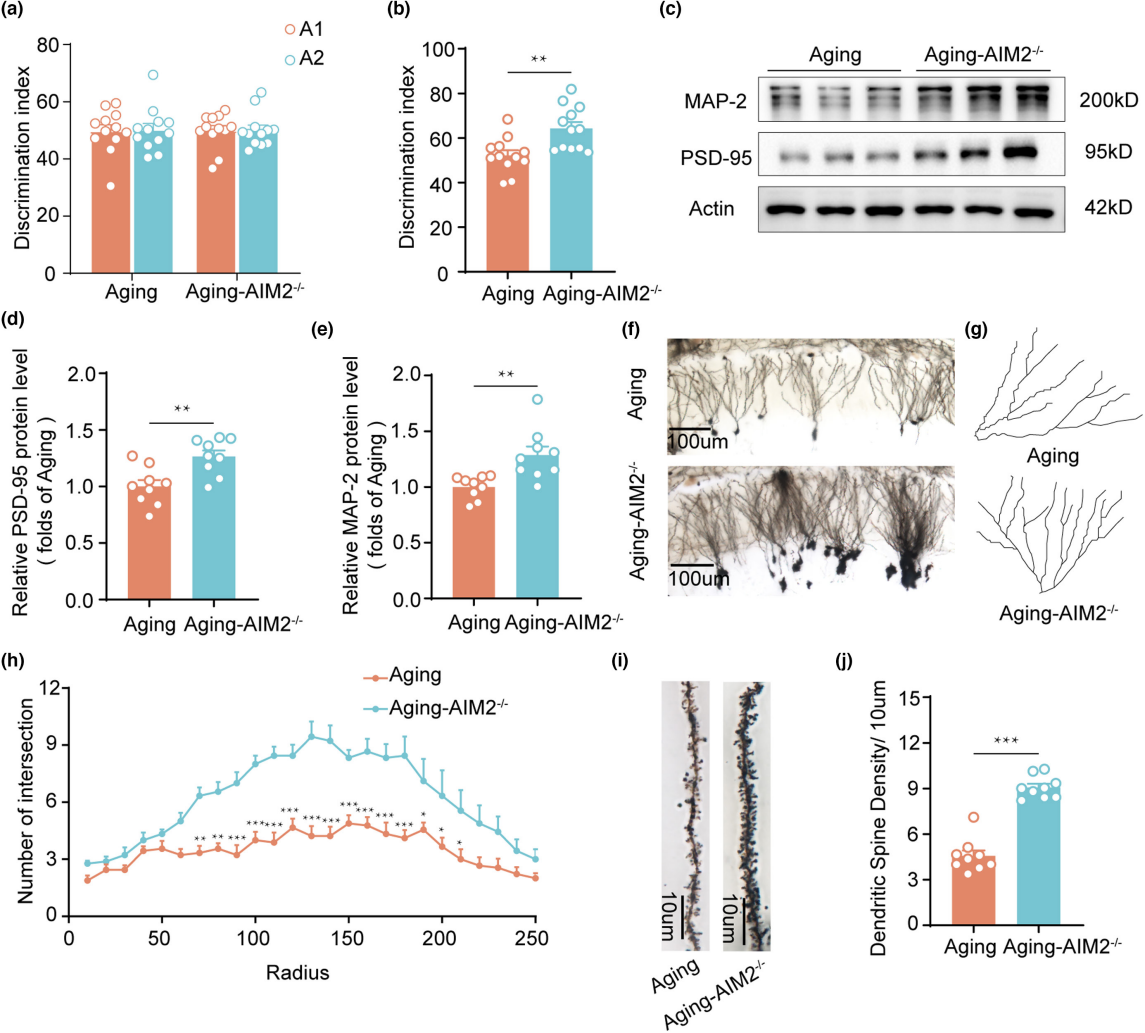
AIM2^−/−^ mice displayed improved cognitive function and ameliorated synapse loss. The time spent exploring two identical objects during the training phase (a) and the displaced object during the test session (b) by 13‐month‐old WT and AIM2^−/−^ male mice in the OPS test was recorded. *n* = 12 for each group. *t*(22) = 3.351, *p* = 0.0029. (c) Representative western blot image of PSD‐95 and MAP‐2 in the DG region in 13‐month‐old WT and AIM2^−/−^ male mice. Western blot analysis of PSD‐95 (d) and MAP‐2 (e) in 13‐month‐old WT and AIM2^−/−^ male mice. β‐Actin was used as a loading control. *n* = 9 for each group. *t*(16) = 3.411, *p* = 0.0036 for PSD‐95; *t*(16) = 3.402, *p* = 0.0036 for MAP‐2. (f) Low‐magnification view of the DG region in 13‐month‐old WT and AIM2^−/−^ male mice. (g) Representative traces of DG neurons. (h) Quantification of the numbers of intersections at different distances from the soma in 13‐month‐old WT (*n* = 3 neurons/mouse, *N* = 3 mice/group) and AIM2^−/−^ male mice (*n* = 3 neurons/mouse, *N* = 3 mice/group). *F*(1,16) = 44.03, *p* < 0.0001. (i) Representative images of dendrite morphology in DG neurons delineated by Golgi staining. (j) Quantitative analysis of the dendritic spine density in 13‐month‐old WT (*n* = 3 spines/mouse, *N* = 3 mice/group) and AIM2^−/−^ male mice (*n* = 3 spines/mouse, *N* = 3 mice/group). *t*(16) = 10.29, *p* < 0.0001. Data were shown as mean ± SEM. Unpaired two‐tailed *t*‐test for b, d, e and j. Two‐way ANOVA followed by Bonferroni's post hoc test for h. *p < 0.05, **p < 0.01, ***p < 0.001.

Since AIM2 expression was elevated in aging mice, we further investigated the effect of AIM2 deletion on memory function. First, we examined whether AIM2 deficiency could alter neural development. The results revealed that AIM2‐KO mice displayed a similar body weight as control mice and showed no global morphological deficits at 2 months of age (Figure S3). Using the open field test to assess motor function and anxiety levels, we found no differences in the mean speed or time spent in the corner or center zone between the aging and aging AIM2^−/−^ groups (Figure S4). Moreover, aging AIM2^−/−^ mice exhibited a significant increase in the discrimination index in the test phase of the object PS test (Figure 2a,b). These results indicated that AIM2 deletion attenuated PS impairment in aging mice.

To elucidate the mechanism by which AIM2 deletion ameliorates cognitive impairment during aging, we further examined the expression of synaptic proteins, and our data showed that the expression levels of PSD‐95 and MAP‐2 were increased in aging AIM2^−/−^ mice compared with aging mice (Figure 2c–e), while there was no difference in the expression of SYN‐1 (Figure S5). Further analysis of neuronal complexity and dendritic spine density by Golgi staining revealed that the dendritic arborizations of granule cells in aging AIM2^−/−^ mice were more complex than those of granule cells in aging mice (Figure 2f–h). Moreover, we counted the number of spines and found a significantly higher dendritic spine density in aging AIM2^−/−^ mice (Figure 2i,j). Taken together, knockout of AIM2 in aging mice ameliorated synapse loss and neuronal impairment, thus rescuing memory function.

### 2.3 Overexpression of AIM2 contributed to impaired synaptic structure and function

**FIGURE 3:**
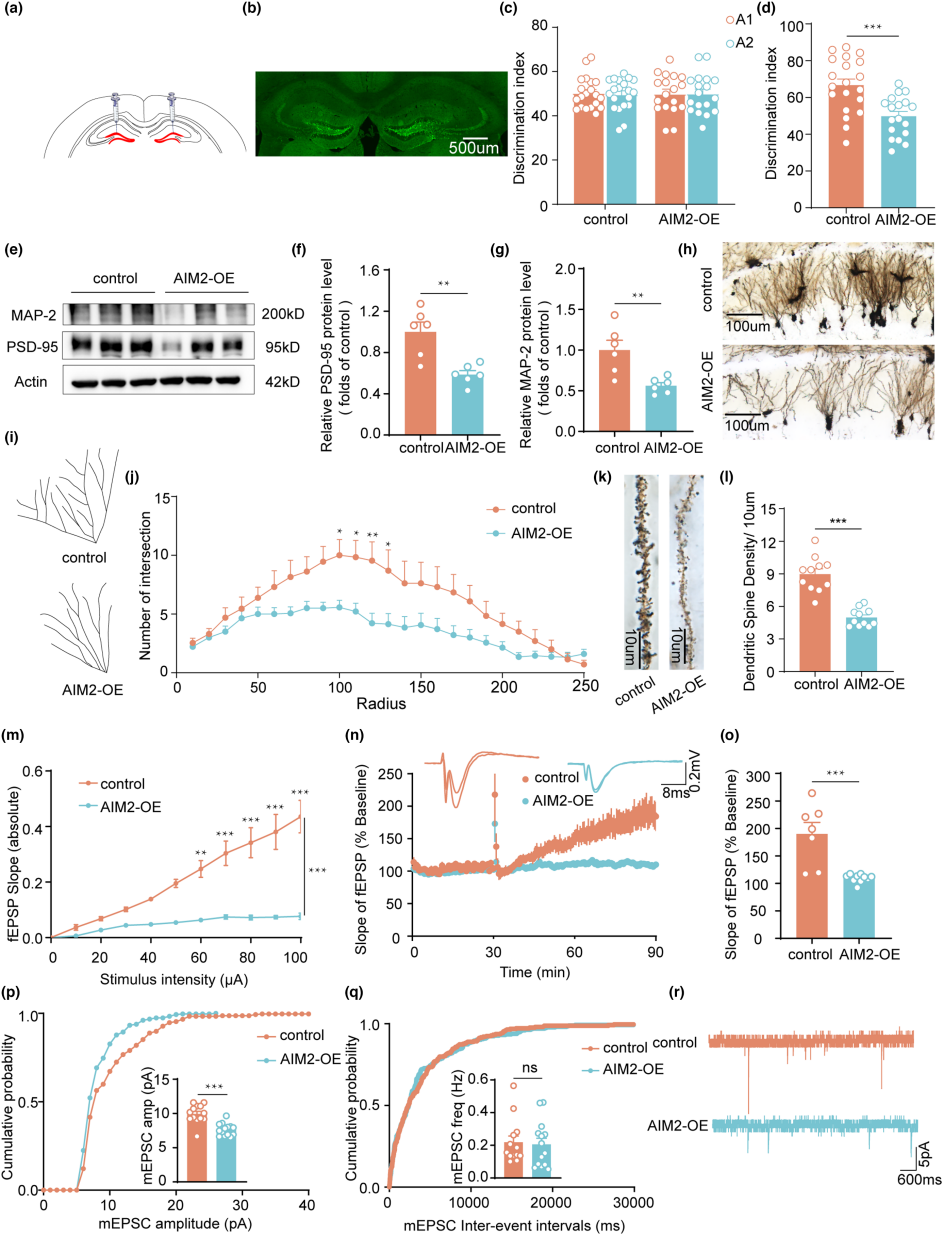
Overexpression of AIM2 in vivo contributed to impaired synaptic structure and function. (a) Schematic of lentivirus microinjection into the DG. (b) Representative images showing the location of EGFP (green) expression. The time spent exploring two identical objects during the training phase (c) and the displaced object during the test session (d) by control and AIM2‐OE mice in the OPS test was recorded. *n* = 18–20 for each group. *t*(36) =3.932, *p* = 0.0004. Western blotting (e) and quantification of PSD‐95 (f) and MAP‐2 (g) protein levels in the DG region of control and AIM2‐OE mice. *n* = 6 for each group. *t*(10) = 4.065, *p* = 0.0023 for PSD‐95; *t*(10) = 3.490, *p* = 0.0058 for MAP‐2. (h) Overview of the DG region in control and AIM2‐OE mice. (i) Neuronal tracing in control and AIM2‐OE mice. (j) Quantification of the numbers of neuronal intersections in control (*n* = 2–3 neurons/mouse, *N* = 5 mice/group) and AIM2‐OE (*n* = 2–3 neurons/mouse, *N* = 5 mice/group) mice by Sholl analysis. *F*(1, 25) = 4.270, *p* = 0.0493. (k) Representative images of dendrite morphology. (l) Quantitative analysis of the mean spine density in control (*n* = 2–3 spines/mouse, *N* = 5 mice/group) and AIM2‐OE (*n* = 2–3 spines/mouse, *N* = 5 mice/group) mice. *t*(22) = 7.368, *p* < 0.0001. (m) The I/O curve of AIM2‐OE mice (*n* = 2–3 slices/mouse, *N* = 4 mice/group) was decreased compared with that of control mice (*n* = 1–3 slices/mouse, *N* = 4 mice/group). *F*(1,12) = 54.81, *p* < 0.0001. (n, o) LTP in the DG region was evaluated in hippocampal slices from control (*n* = 1–3 slices/mouse, *N* = 4 mice/group) and AIM2‐OE (*n* = 2–3 slices/mouse, *N* = 4 mice/group) mice. The slope of the regression line decreased markedly after overexpression of AIM2. *t*(15) = 4.631, *p* = 0.0003. Mean mEPSC amplitude (p) and frequency (q) in DG neurons of control (*n* = 3–5 neurons/mouse, *N* = 3 mice/group) and AIM2‐OE (*n* = 3–5 neurons/mouse, *N* = 3 mice/group) mice. *t*(25) = 5.231, *p* < 0.0001 for amplitude; *t*(25) = 0.2409, *p* = 0.8116 for frequency. (r) Representative traces of mEPSC recordings in DG hippocampal acute slices from control and AIM2‐OE mice. Data were shown as mean ± SEM. Unpaired two‐tailed *t*‐test for d, f, g, l, o, p and q. Two‐way ANOVA followed by Bonferroni's post hoc test for j and m. *p < 0.05, **p < 0.01, ***p < 0.001; ns no significance.

To further investigate whether AIM2 is a critical regulator in impaired PS at an early state of senescence, we injected an AIM2‐overexpressing lentivirus (AIM2‐OE) or a control lentivirus into the DG of 8‐week‐old C57BL/6 mice (Figure 3a,b). We confirmed that the AIM2 expression level increased in the DG after viral infection by RT–PCR and western blotting (Figure S6A–C). Next, we conducted behavioral tests and found no differences in motor activity and anxiety performance between groups (Figure S6D–G). In the object PS test, the time spent exploring the object in the novel location by AIM2‐OE mice was similar to that spent by 13‐month‐old mice and was markedly reduced compared to that spent by control mice (Figure 3c,d; Figure S7A,B). Collectively, these results revealed that impaired PS occurred due to elevated expression of AIM2 in mice.

Next, we explored the impact of AIM2 on the structural and functional properties of synapses. Decreased expression of PSD‐95 and MAP‐2 in AIM2‐OE mice versus control mice was detected by western blot analysis (Figure 3e–g), while the expression of SYN‐1 was not significantly altered (Figure S8). In addition, we performed Golgi staining and Sholl analysis on neurons in the DG. As expected, Sholl analysis revealed that the morphological complexity of neurons in the DG was significantly lower in the AIM2‐OE group than in the control group (Figure 3h–j; Figure S7C–E), as was the dendritic spine density (Figure 3k,l; Figure S7F,G).

Then, the basal synaptic transmission and synaptic plasticity of hippocampal neurons were examined. First, to characterize basic synaptic transmission in AIM2‐OE mice, the input–output (I–O) relationship was measured. As the stimulus intensity increased, the AIM2‐OE mice showed a significantly decreased field excitatory postsynaptic potential (fEPSP) slope compared with the control mice (Figure 3m), suggesting defective synaptic transmission. Second, long‐term potentiation (LTP) measurements were performed, and AIM2‐OE mice exhibited lower averages of normalized fEPSP slope than control mice (Figure 3n,o). Overexpression of AIM2 in the DG region did not result in significant changes in basal synaptic transmission or synaptic plasticity in the CA1 region (Figure S9). Miniature excitatory postsynaptic currents (mEPSCs) of granule cells were also recorded in the DG region using a whole‐cell patch clamp. The results showed that neurons from AIM2‐OE mice displayed a lower mEPSC amplitude, while the frequency was unaffected (Figure 3p–r), indicating impaired postsynaptic function. These results suggested that overexpression of AIM2 resulted in the disruption of synaptic structures and plasticity in the DG region.

### 2.4 AIM2 regulated microglial phagocytic activity and synapse engulfment

**FIGURE 4:**
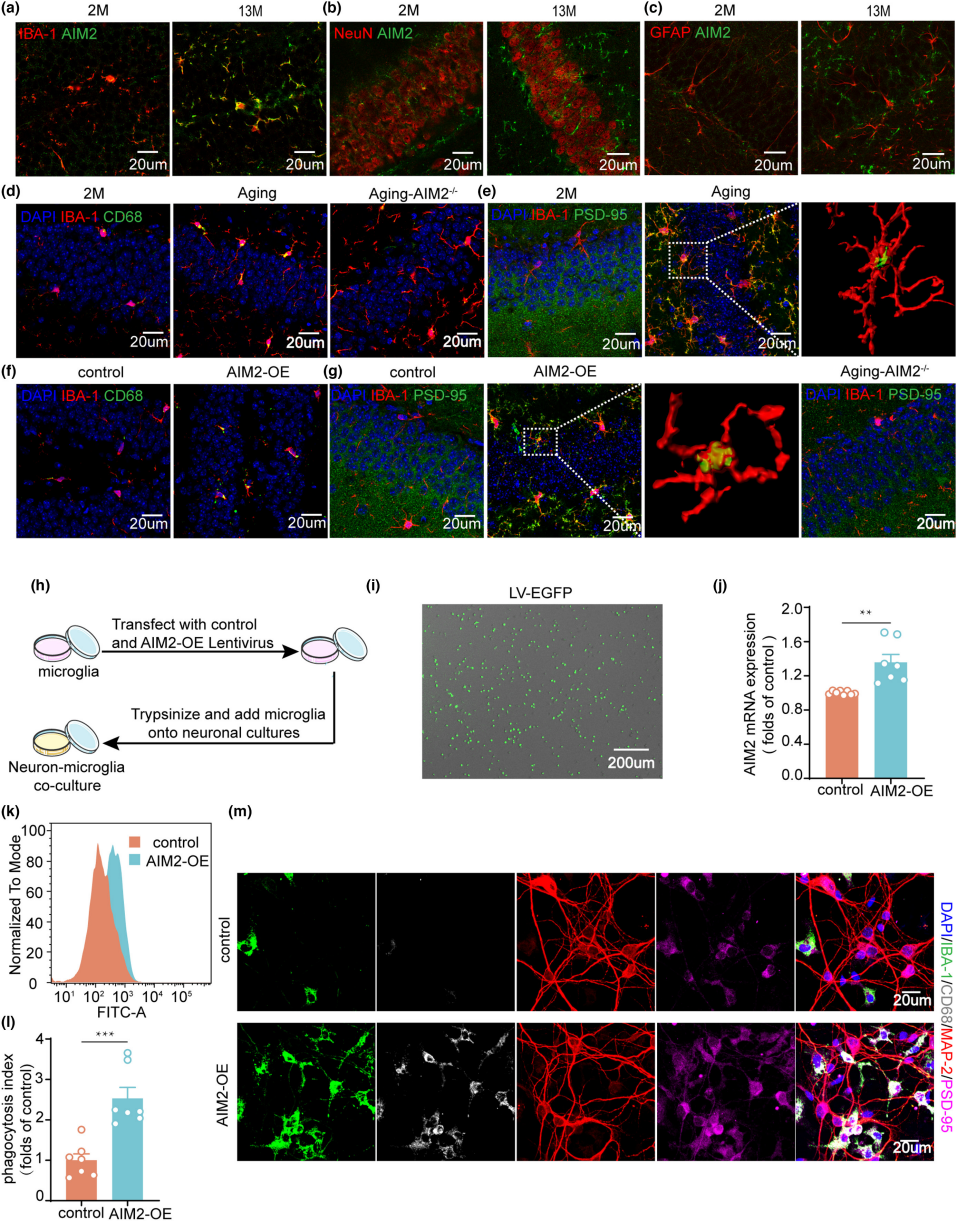
AIM2 regulated microglial phagocytic activity and synapse engulfment. Double immunofluorescence staining of AIM2 (green) with IBA‐1 (red) (a), NeuN (red) (b) and GFAP (red) (c) in the DG of 2‐ and 13‐month‐old male mice. (d) High levels of CD68 (green) immunoreactivity in IBA‐1^+^ (red) microglia were detected in 13‐month‐old male mice. (e) Confocal images showing the presence of PSD‐95^+^ (green) puncta around IBA‐1^+^ (red) microglia and the corresponding 3D reconstructions. (f) Immunostaining for IBA‐1 (red) and CD68 (green) in the DG region of control and AIM2‐OE mice suggested that AIM2 overexpression induced high levels of CD68 (green) immunoreactivity in IBA‐1‐positive (red) microglia. (g) Confocal images showed IBA‐1 (red) coexpression with PSD‐95 (green), and 3D reconstruction demonstrated that IBA‐1^+^ microglia in AIM2‐OE mice engulf more synaptic elements. (h) Schematic diagram of microglia and neuron coculture. The overexpression efficiency of the lentivirus was determined through EGFP‐positive (green) cells observed via fluorescence microscopy (i) and AIM2 levels via RT–PCR (j). *n* = 8 for the control group, *n* = 7 for the AIM2‐OE group. *t*(13) = 4.104, *p* = 0.0012. Flow cytometric analysis (k) and quantification (l) of microglial phagocytic activity for fluorescent microspheres. *n* = 7 for each group. *t*(12) = 4.876, *p* = 0.0004. (m) Representative confocal images showing the localization of IBA‐1 (green), CD68 (gray), MAP‐2 (red) and PSD‐95 (magenta) in microglia transfected with the control or AIM2‐OE lentivirus. Data were shown as mean ± SEM. Unpaired two‐tailed *t*‐test for j and l. **p < 0.01, ***p < 0.001.

Our immunostaining results showed AIM2 expression in microglia was increased in the hippocampus of older individuals compared to young individuals (Figure S10). Furthermore, we found that in aging mice, AIM2 expression was increased in microglia but not neurons or astrocytes in the DG region (Figure 4a–c; Figure S11), suggesting that AIM2 may play a key role in regulating microglia, thereby mediating cognitive dysfunction during aging. Microglia, scavenging cells in the CNS, have been shown to be important in synaptic remodeling under physiological and pathological conditions (Hong et al., 2016; Nguyen et al., 2020).

To assess the phagocytic capacity of microglia, we utilized IBA‐1 to label microglia and the phagocytic marker CD68 to label phagocytic cells. The results indicated that the number of IBA‐1^+^/CD68^+^ microglia increased in aging mice compared with 2‐month‐old mice, while the knockout of AIM2 decreased the number of IBA‐1^+^/CD68^+^ microglia (Figure 4d). Additionally, microglial phagocytosis of synapses was evaluated by immunostaining, and more PSD‐95 immunosignals were colocalized with microglia in aging mice than in 2‐month‐old and aging AIM2^−/−^ mice. Internalization of PSD‐95 within microglia was shown in 3D reconstruction of confocal *z* stacks (Figure 4e). To further investigate whether overexpression of AIM2 enhanced microglial synapse elimination, we performed immunofluorescence staining and observed significantly more phagocytic IBA‐1^+^/CD68^+^ microglia (Figure 4f) and more PSD‐95 puncta in IBA‐1^+^ microglia (Figure 4g) in AIM2‐OE mice, while there was markedly less colocalization of SYN‐1 with IBA‐1+/CD68+ microglia in AIM2‐OE mice and Aging mice (Figure S12). Taken together, these results confirmed an essential role for AIM2 in microglial phagocytosis of synapses.

To further assess the effects of AIM2 on synaptic structures, primary microglia were transfected with a control or AIM2‐OE lentivirus and subsequently cocultured with primary neurons (Figure 4h). The increased EGFP expression and AIM2 level, measured by fluorescence microscopy and RT–PCR analysis, respectively, confirmed the transfection efficiency (Figure 4i,j). After being cultured in the presence of fluorescence‐labeled microspheres, flow cytometry results showed that primary microglia overexpressing AIM2 displayed enhanced phagocytic activity (Figure 4k,l). In the neuron–microglia coculture system, we observed that more PSD‐95^+^ and MAP‐2^+^ puncta were localized in IBA‐1+ CD68+ microglia in the AIM2‐OE group than in the control group (Figure 4m). These results suggested that overexpression of AIM2 in microglia correlated with increased synapse engulfment in vitro.

### 2.5 AIM2 regulated microglial synaptic engulfment via the complement pathway

**FIGURE 5:**
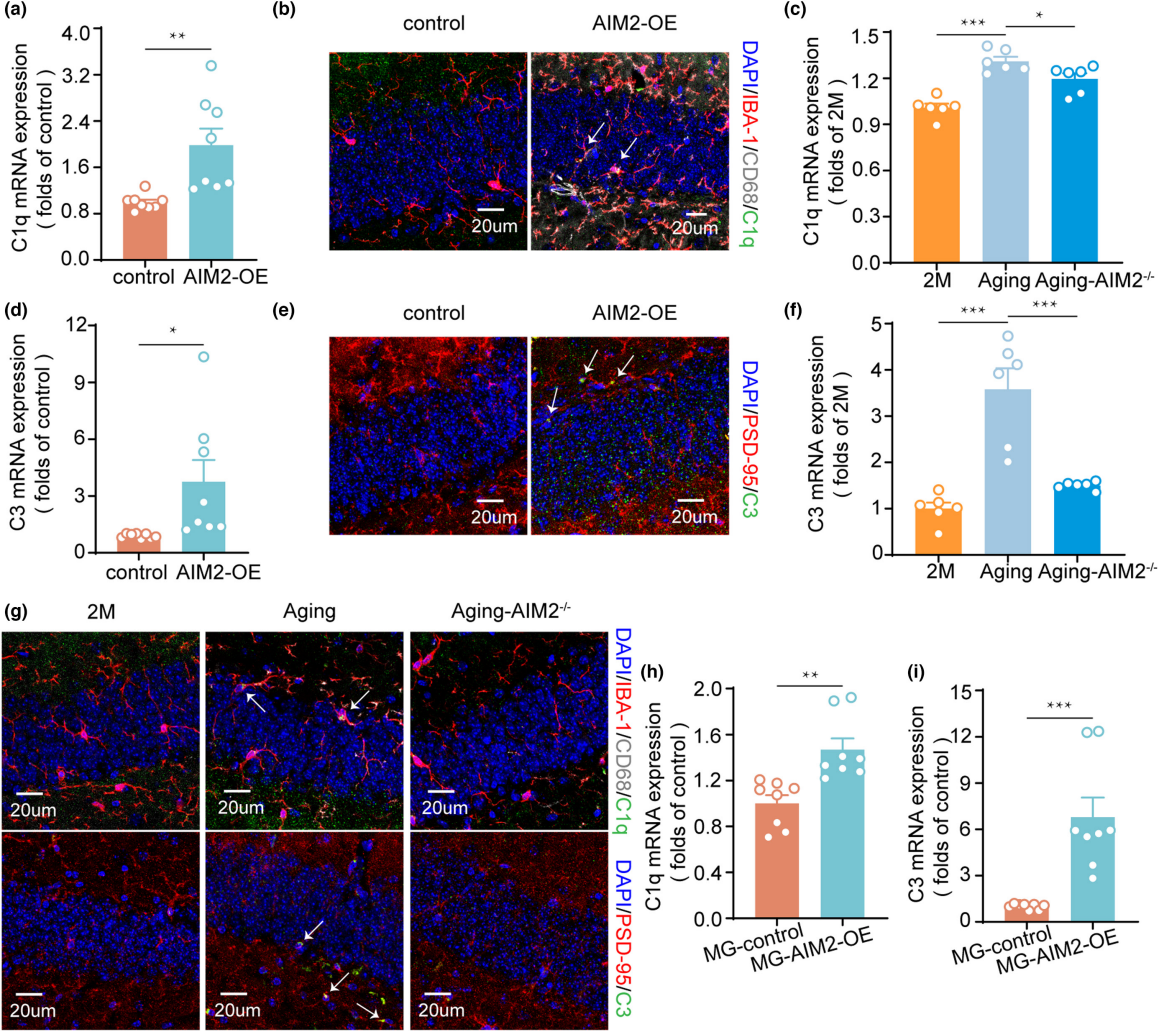
AIM2 regulated microglial synaptic engulfment via the complement pathway. (a) The mRNA level of C1q in the DG region of control and AIM2‐OE mice via quantitative RT–PCR. *n* = 8 for each group. *t*(14) = 3.411, *p* = 0.0042. (b) Colocalization of C1q (green) with IBA‐1 (red) and CD68 (gray) in the hippocampal DG of control and AIM2‐OE mice. (c) RT–PCR analysis of C1q mRNA in the 2‐month‐old, 13‐month‐old WT and 13‐month‐old AIM2^−/−^ group. *n* = 6 for each group. *F*(2,15) = 0.2174, 13‐month‐old WT group vs. 2‐month‐old group: *p* < 0.0001, 13‐month‐old AIM2^−/−^ vs. 13‐month‐old WT group: *p* = 0.0380. (d) The mRNA level of C3 in the DG region of control and AIM2‐OE mice by quantitative RT–PCR. *n* = 8 for each group. *t*(14) = 2.469, *p* = 0.0270. (e) Colocalization of C3 (green) with PSD‐95 (red) in the hippocampal DG of control and AIM2‐OE mice. (f) RT–PCR analysis of C3 mRNA in the 2‐month‐old, 13‐month‐old WT and 13‐month‐old AIM2^−/−^ group. *n* = 6 for each group. *F*(2,15) = 3.982, aging group versus 2 m group: *p* < 0.0001, aging group vs. aging AIM2^−/−^ group: *p* = 0.0002. (g) Colocalization of C1q (green) with IBA‐1 (red) and CD68 (gray), C3 (green) with PSD‐95 (red) in the 2‐month‐old, 13‐month‐old WT and 13‐month‐old AIM2^−/−^ group. The mRNA expression levels of C1q (h) and C3 (i) were measured in microglia transfected with the control or AIM2‐OE lentivirus and normalized to GAPDH mRNA expression. *n* = 8 for each group. *t*(14) = 3.849, *p* = 0.0018 for C1q; *t*(14) = 4.529, *p* = 0.0005 for C3. Data were shown as mean ± SEM. Unpaired two‐tailed *t*‐test for a, d, h and i. One‐way ANOVA followed by Dunnett's post hoc test for c and f. *p < 0.05, **p < 0.01, ***p < 0.001.

The classical complement cascade has been shown to participate in synaptic pruning mediated by microglia (Hong et al., 2016) and age‐associated cognitive impairment (Shi et al., 2015). Thus, we wanted to explore whether the complement pathway is engaged in microglia‐mediated synaptic engulfment in aging mice. We first examined the distribution and relative expression of C1q and C3 in AIM2‐OE and control mice. Notably, the C1q level was higher in the AIM2‐OE group (Figure 5a), and the localization of C1q with IBA‐1^+^ CD68^+^ cells demonstrated that C1q was localized mainly in microglia (Figure 5b; Figure S13A). In addition, as expected, the level of C1q in the aging group was markedly increased compared with that in the 2‐month‐old group, and AIM2 deficiency decreased the C1q level (Figure 5c). Similarly, the increase in C3 expression was accompanied by the increased colocalization of C3 and PSD‐95 in AIM2‐OE mice (Figure 5d,e; Figure S13B). Furthermore, RT–PCR results showed that C3 expression in the 2‐month‐old group, aging group and aging AIM2^−/−^ group followed the same pattern as C1q expression (Figure 5f). C1q expression in IBA‐1+/CD68+ microglia and localization of C3 in hippocampal synapses were increased in aging mice compared with the 2‐month‐old mice, whereas knockout of AIM2 partially reversed these effects, which were detected only in the DG region of hippocampus (Figure 5g and Figures S14–S17). Additionally, we found C3 showed less colocalization with SYN‐1 in AIM2‐OE mice and Aging mice (Figure S18), indicating that C3 was specifically deposited on postsynaptic elements. Consistent with the in vivo experiments, overexpression of AIM2 in primary microglia increased the expression levels of C1q and C3 (Figure 5h,i). Overall, these results indicated that the complement pathway was necessary for synaptic pruning by microglia, and this regulation was specifically targeted to postsynaptic densities.

### 2.6 C3aR antagonist improved cognitive deficits and rescued synapse density in AIM2‐overexpressing and aging mice

**FIGURE 6:**
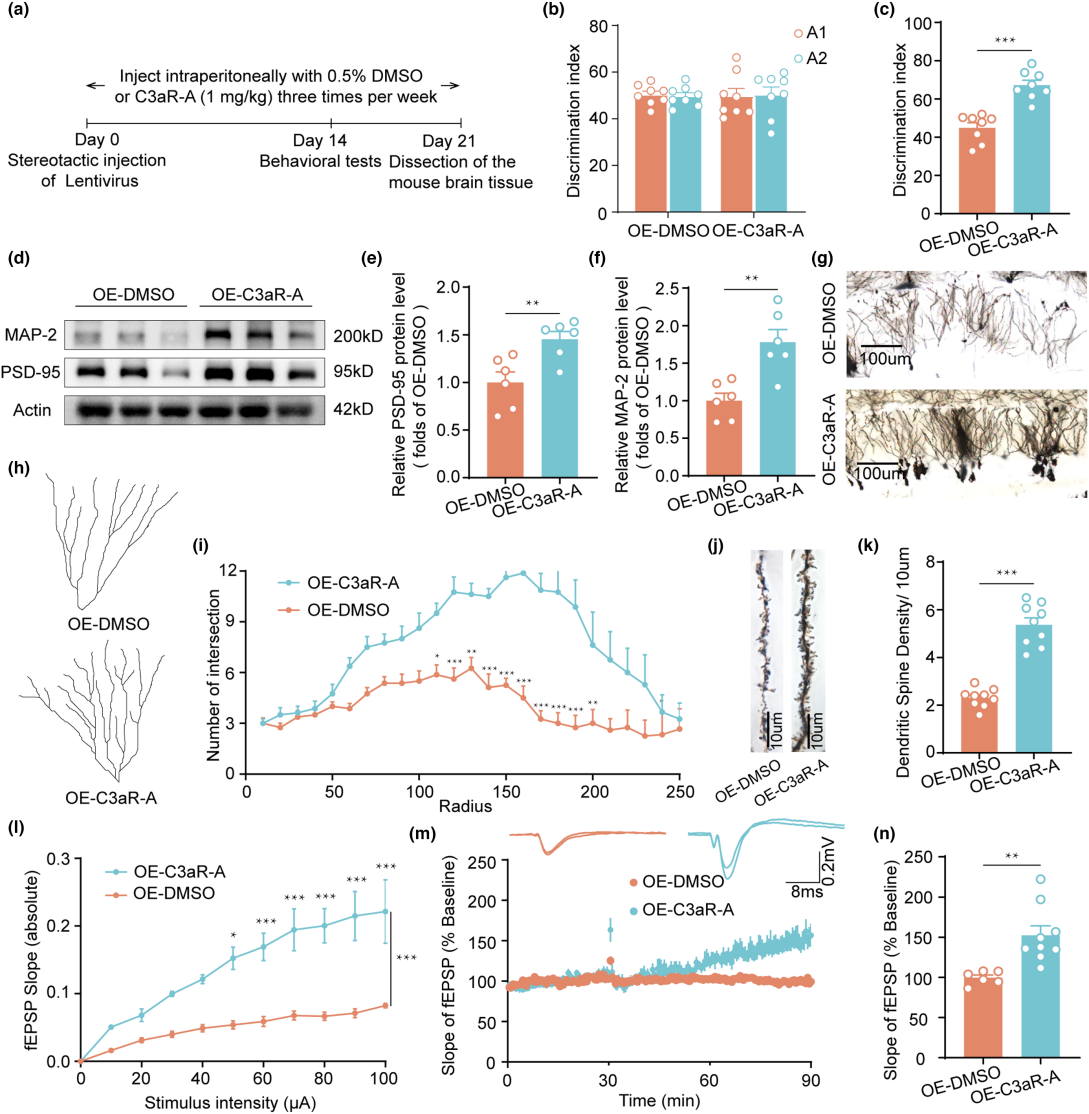
C3aR antagonist improves cognitive deficits and rescues synapse density in AIM2‐overexpressing and aging mice. (a) Flow chart of the experimental design. The time spent exploring two identical objects during the training phase (b) and the displaced object during the test session (c) in the OPS test of OE‐DMSO and OE‐C3aR‐A mice were recorded. *n* = 8 for each group. *t*(14) = 6.058, *p* < 0.0001. (d) PSD‐95 and MAP‐2 protein expression levels in the DG were analyzed using an immunoblot assay. The protein expression levels of PSD‐95 (e) and MAP‐2 (f) were normalized to that of β‐actin as a loading control. *n* = 6 for each group. *t*(10) = 3.310, *p* = 0.0079 for PSD‐95; *t*(10) = 3.966, *p* = 0.0027 for MAP‐2. (g) Overview of the DG region in OE‐DMSO and OE‐C3aR‐A mice. (h) Neuron tracing in OE‐DMSO and OE‐C3aR‐A mice. (i) Quantification of the number of neuronal intersections in OE‐DMSO (*n* = 2–3 neurons/mouse, *N* = 3 mice/group) and OE‐C3aR‐A (*n* = 2–3 neurons/mouse, *N* = 3 mice/group) mice by Sholl analysis. *F*(1,14) = 31.91, *p* < 0.0001. (j) Representative images of dendrite morphology. (k) Quantitative analysis of the average spine density in OE‐DMSO (*n* = 3 spines/mouse, *N* = 3 mice/group) and OE‐C3aR‐A (*n* = 3 spines/mouse, *N* = 3 mice/group) mice. *t*(16) = 9.533, *p* < 0.0001. (l) The field EPSP (fEPSP) amplitude was increased in OE‐C3aR‐A mice (*n* = 3 slices/mouse, *N* = 3 mice/group) compared with OE‐DMSO mice (*n* = 3 slices/mouse, *N* = 3 mice/group). *F*(1,16) = 27.14, *p* < 0.0001. (m, n) LTP induced by HFS was evaluated in the hippocampal DG following C3aR inhibition in AIM2‐OE mice (*n* = 3 slices/mouse, *N* = 3 mice/group) and compared with that in OE‐DMSO mice (*n* = 1–3 slices/mouse, *N* = 3 mice/group). *t*(13) = 3.540, *p* = 0.0036. Data were shown as mean ± SEM. Unpaired two‐tailed *t*‐test for c, e, f, k and n. Two‐way ANOVA followed by Bonferroni's post hoc test for i and l. *p < 0.05, **p < 0.01, ***p < 0.001.

Given the important role of C3‐C3aR signaling in synaptic function (Lian et al., 2015), we wanted to examine the effects of C3aR antagonists (C3aR‐A) on memory functions in AIM2‐OE or aging mice and performed behavioral tests (Figure 6a; Figure S20A). C3aR‐A treatment did not affect the general locomotor activity or anxiety levels of AIM2‐OE and aging mice (Figures S19 and S20B‐E) but significantly increased the discrimination index in the object PS test (Figure 6b,c; Figure S20F‐G). Overall, these data demonstrated that C3aR‐A treatment ameliorated memory impairment in AIM2‐OE and aging mice.

Similarly, we examined the effects of C3aR‐A on synaptic proteins, synaptic structure, and transmission in the DG region of AIM2‐OE and aging mice. As evident from the western blot analyses, C3aR‐A treatment resulted in increased levels of PSD‐95 and MAP‐2 (Figure 6d–f; Figure S20H‐J). Quantitative analysis of the effects of C3aR‐A treatment on neuronal morphology and dendritic spine revealed a significant increase in dendritic complexity (Figure 6g–i) and spine density (Figure 6j,k) in the DG. Furthermore, basic synaptic transmission and long‐term synaptic plasticity were enhanced by C3aR inhibition in AIM2‐OE mice (Figure 6l–n). Taken together, these results indicated that C3aR‐A treatment improved synaptic and memory functions in AIM2‐OE mice.

## 3 DISCUSSION

In this study, we confirmed that PS behavior was impaired in aging mice and that this behavior was closely related to synaptic loss in the DG region of the hippocampus. Our results revealed that AIM2 expression was increased in aging mice and that upregulation of AIM2 in the DG of wild‐type (WT) mice could impair PS behavior and induce synaptic damage similar to the observed patterns in aging mice. AIM2 deletion ameliorated synaptic damage in the DG and PS behavior impairment in aging mice. Furthermore, increased AIM2 levels promoted microglial phagocytosis of synapses in a complement‐dependent manner. Administration of a C3aR inhibitor effectively inhibited AIM2 overexpression‐induced microglial activation in synaptic pruning, ameliorated impaired synaptic plasticity and alleviated dysfunctional PS behavior. Taken together, our results suggest a critical role of AIM2 in mediating synaptic plasticity and cognitive behavior in aging by regulating microglial phagocytosis via the complement pathway. Our study shows a new role for AIM2 in regulating aging‐related synaptic loss and cognitive dysfunction.

Aging is a natural process associated with cognitive decline and is closely associated with serious debilitating neurodegenerative diseases, such as AD (Miller et al., 2008). The neuroanatomical basis for age‐related cognitive functional impairment has not been established definitively; however, it likely depends on the maintenance of synaptic contacts and does not involve a loss of neurons in the early stage of aging (Hof & Morrison, 2004; Yeoman et al., 2012). Moreover, cognitive decline associated with aging is closely associated with hippocampal alterations (Bettio et al., 2017). The DG in the hippocampus has been proposed to play an important role in the formation of PS, which has been associated with NMDA receptor expression in the DG (Deng et al., 2010; Kannangara et al., 2015). Our results showed significant synaptic loss in the DG of aging mice, and AIM2 regulated neuronal synaptic structure and plasticity and affected PS function.

Previous studies have reported that AIM2 affects cognitive function in diseases such as AD, stroke and vascular dementia. Our previous research showed that AIM2 protein levels were significantly upregulated in the hippocampus of AD mice and that AIM2 deletion promoted neuroplasticity and spatial memory in mice (Chen et al., 2019). It has been demonstrated that AIM2 regulates neuronal morphology, including dendritic growth and axon extension, and influences mouse behaviors, including anxiety and memory (Wu et al., 2016). In addition, AIM2‐mediated inflammation and pyroptosis likely aggravate poststroke cognitive impairment, and AIM2 knockout significantly improves cognitive function and reverses the decrease in hippocampal volume in stroke mice (Kim et al., 2020). Furthermore, activation of the AIM2 inflammasome substantially contributes to the pathophysiology of chronic cerebral hypoperfusion‐induced brain injury and related cognitive impairment (Poh et al., 2021). However, there is no previous study on the effect of AIM2 on aging‐related cognitive dysfunction. In this study, we first demonstrated that AIM2 mediated aging‐related cognitive dysfunction by regulating microglial phagocytosis of neuronal synapses.

AIM2 is widely expressed in the brain, with the highest levels occurring in microglia under physiological conditions (Zhang et al., 2014). Increased oxidative stress and neuroinflammation, reduced neurogenesis and synaptic plasticity, and altered intracellular signaling and gene expression in the aging hippocampus are considered to be associated with age‐related cognitive decline (Bettio et al., 2017). Among these, neuroinflammation during aging inevitably affects the functional properties of microglia, and altered microglial function is also a hallmark of brain aging (Brawek et al., 2021; Ransohoff, 2016). During aging, microglia are gradually activated and exhibit severe functional abnormalities, such as increased production of proinflammatory cytokines and reactive oxygen species (Harry, 2013). Many previous studies have reported that AIM2 controls microglial inflammation (Ma et al., 2021; Zhang et al., 2020). Our previous study revealed that AIM2 deletion inhibited microglial infiltration 48 h after stroke and protected against ischemic brain injury (Zhang et al., 2020). Our data confirmed that AIM2 overexpression activated microglial phagocytosis and proinflammatory cytokines, such as IL‐1β and IL‐18 (data not shown), and that AIM2 deletion could effectively inhibit microglial phagocytosis of synapses and increase proinflammatory cytokine levels in the DG of aging mice.

However, there is no direct evidence that AIM2 regulates the phagocytic function of microglia thus far, and the underlying mechanism is also unclear. A study of AIM2 deletion in a mouse model of AD indicated that the lack of Aim2 protein function reduced microglial activation and Aβ deposition in the brain (Wu et al., 2017). For this demonstration, a review speculated that knockout of AIM2 affected microglial TREM2 receptor expression by increasing IFN‐β and activating the IFN response in microglia and that the TREM receptor could regulate microglial function, including phagocytosis of Aβ deposits (Choubey, 2019; Choubey & Panchanathan, 2020; Nazmi et al., 2019). In our study, we revealed that AIM2 regulates microglial phagocytosis of neuronal synapses via the complement pathway, and this regulation is specifically targeted to postsynaptic densities, which has not been reported in previous studies.

The complement system is an important part of the eat‐me signaling system and is closely related to abnormal phagocytosis after microglial activation (Stevens et al., 2007). Complement proteins can be activated by the classical pathway, lectin pathway and alternative pathway (Zarantonello et al., 2023). The complement protein C1q is the initiating protein of the classical complement system, and the downstream molecule complement protein C3 is the central molecule of the complement system and is the point of convergence of all its activation pathways (Gasque, 2004; Zarantonello et al., 2023). Deposited C3 can activate C3 receptors on microglia directly, thus triggering synapse elimination by phagocytosis (Stevens et al., 2007). It has been shown that during the aging process, the progressive accumulation of complement C1q and C3 in the hippocampus promotes cognitive decline and memory impairments (Shi et al., 2015; Stephan et al., 2013). Another study indicated that gene knockout or inhibition of the C3/C3R signaling system reduced the abnormal phagocytosis of synapses by microglia and delayed the process of virus‐induced synapse loss and cognitive impairment (Lui et al., 2016; Vasek et al., 2016). Our results confirmed that the C1q and C3 levels were increased in aging mice. Furthermore, we demonstrated that overexpression of AIM2 resulted in upregulated transcription of C1q and C3. Inhibiting C3aR could effectively mitigate the synaptic loss, synaptic plasticity impairment and PS dysfunction induced by AIM2 overexpression, and administration of a C3aR inhibitor could ameliorate PS dysfunction in aging mice. However, the mechanism by which AIM2 regulates the complement system remains unclear and requires further investigation.

In summary, this study indicates that the increased expression of AIM2 mediates abnormal synapse phagocytosis by microglia through the complement pathway, resulting in a decrease in synaptic plasticity in the DG region and impaired PS behavior in aging mice. These findings reveal a novel role for AIM2 in regulating microglial synapse phagocytosis during aging. These findings provide potential targets for ameliorating age‐associated cognitive dysfunction.

## 4 EXPERIMENTAL PROCEDURES

### 4.1 Mouse models and treatment

And 2‐ and 13‐month‐old C57BL/6 male mice, as well as WT and AIM2‐KO male mice were obtained from the Model Animal Research Center of Nanjing University, Ziyuan Biotechnology (Hangzhou, China), Wukong Biotechnology (Nanjing, China) and GemPharmatech company (Nanjing, China). Overexpression lentivirus for AIM2 as well as control lentivirus was constructed by GeneChem (Shanghai, China). 8‐week‐old C57BL/6 male mice were anesthetized and placed in a stereotaxic frame for bilateral injection (1.0 μL) of LV‐AIM2 or LV‐con into the hippocampal DG region (1.94 mm posterior to bregma, 1.25 mm lateral to midline and 1.88 mm below the skull surface). And 13‐month‐old male mice or virus‐injected mice were treated with DMSO or C3aR‐A (1 mg/kg) by intraperitoneal injection three times a week (Mondays, Wednesdays, and Fridays) for the following weeks. Behavioral experiments and electrophysiological recordings were performed 2 weeks after virus injection. All experiments related to animals were approved by the Animal Care Committee of Nanjing University.

### 4.2 Postmortem samples

Postmortem human hippocampus was obtained from the Chinese Brain Bank Center (CBBC, http://cbbc.scuec.edu.cn; Wuhan, China). All human experiments were approved by the Research Ethics Committee of South‐Central University for Nationalities (2021‐scuec‐034).

### 4.3 Primary cell culture, neuron–microglia co‐culture and treatment

#### 4.3.1 Primary microglial culture

Primary microglia were isolated from mixed glia established from the cerebral cortices of newborn C57/BL6J mice as previously described (Zhang et al., 2020). After 10–12 days in culture, floating microglia on the mixed primary glia layer were isolated through shaking the flasks and were seeded into 12‐ or 24‐well plates for approximately 36 h. Cell culture media were supplemented with 10% fetal bovine serum (FBS, Biological Industries) and 1% penicillin/streptomycin and grown in humidified 5% CO_2_ incubator at 37°C. Primary cultured microglia were transduced with LV‐AIM2 or LV‐con (at a multiplicity of infection of 10) 36 h after seeding. Six hours after transfection, the virus‐containing medium was replaced with complete medium and incubated for 2 days neuron–microglia co‐culture.

#### 4.3.2 Primary neuron culture

Primary cortical neurons were prepared from C57/BL6J mice embryos at E15‐17 as previously described (Tao et al., 2021). Neurons were seeded in neurobasal medium supplemented with 25 nM glutamine and B27 (Invitrogen) at 37°C in a humidified incubator with 5% CO_2_.

#### 4.3.3 Neuron–microglia co‐culture

Two days post infection, primary microglia were trypsinized for 5 min and the digestion was stopped by DMEM containing 10% FBS. After 800 rpm centrifuging for 5 min, microglia were resuspended in NbActiv4 media. In co‐culture, microglia were cultured at a 1:3 ratio with neuron for 24 h in confocal dishes. Co‐cultures were then washed twice with phosphate‐buffered saline (PBS) and fixed in 4% paraformaldehyde (PFA) for further analysis.

### 4.4 Behavioral experiments

#### 4.4.1 Open field

To evaluate motor function and anxiety of the treated mice, we performed open field. The experimental animals were allowed to acclimatize to the environment for 1 h before experiments. The test was conducted in a 48 cm × 48 cm × 36 cm open field box that was divided into 16 identical squares. We defined the four corner squares as corner zone and the four middle squares as center zone. The open field box was cleaned with 75% ethanol after testing of each mouse to minimize odour cues. For each mouse, we quantified mean locomotor speed and time spent in the center and corner zone with ANY‐maze software.

#### 4.4.2 Pattern separation

The PS task was conducted in a nontransparent box measuring 30 × 30 × 45 cm high. The experimental animals were habituated to the testing box for 10 min/day for three consecutive days before testing. During the training phase, mice were exposed to two identical objects placed symmetrically and allowed to explore for 10 min. One hour later, mice were allowed to explore the objects for 5 min with one of the objects switched to a novel location during the test session. Mouse behavior was recorded with a video tracking system. The discrimination index calculated by dividing the time exploring the newly positioned object by the total time was used to measure cognitive function.

#### 4.4.3 Morris water maze

The MWM test was performed to examine the spatial learning and memory of animals. The water maze (120 cm diameter) consisted of a circular pool and an escape platform hidden 1 cm underneath the water surface. During training, the mice were trained to locate the escape platform in a fixed location within 60 s for five consecutive days. If the mouse was unable to find the platform within 60 s, it was guided onto the platform and allowed a rest on the platform for 30 s. The latency during the training stage was assessed with ANY‐maze software (Stoelting Co.). The mice were allowed to search for 60 s during the probe trial with the platform removed, and then the platform crossings, latency to the platform, time spent in target quadrant and swimming speed were measured.

### 4.5 Quantitative real‐time PCR

Total RNA was isolated from primary microglia and the treated mice using TRIzol reagent (Invitrogen), and then cDNA were synthesized using the PrimeScript RT Reagent kit (Takara). Quantitative Real‐Time PCR was performed on a Step One Plus PCR system (Applied Biosystems) using a SYBR Green kit (Applied Biosystems). The gene expression levels were normalized relative to glyceraldehyde‐3‐phosphate dehydrogenase (GAPDH). The primer sequences were as follows:

#### 4.5.1 Sequences of primers for quantitative RT–PCR

**Table:** 

Gene	Primer
GAPDH	F: GCCAAGGCTGTGGGCAAGGT R: TCTCCAGGCGGCACGTCAGA
AIM2	F: CTCAGGAAGGAAGACAAGA R: GATTCAACATCAACCACAAC
C1q	F: CACCGTGCTTCAGCTGCGACGAG R: TTGCGGGGTCCTTTTCGATCCAC
C3	F: ACTGTGGACAACAACCTACTGC R: GCATGTTCGTAAAAGGCTCGG

### 4.6 Western blotting

Tissue lysate was prepared and subjected to western blotting as previously described (Tao et al., 2021). The membranes were incubated with the following primary antibodies at 4°C overnight: rabbit anti‐MAP‐2 (1:1000, Bioworld, BS3487), mouse anti‐PSD‐95 (1:1000, Abcam, ab2723), rabbit anti β‐actin (1:1000, Bioworld, AP‐0060), rabbit anti‐AIM2 (1:500, Abcam, ab119791) and rabbit anti‐SYN‐1 (1:1000, Abcam, ab64581). After washed with 0.1% Tris‐buffered saline with Tween 20 three times (10 min each), the membranes were incubated with the HRP‐conjugated secondary antibodies (1:5000) for 2 h at room temperature. The color detection was carried out with an ECL Western Blotting Detection kit (Millipore), and quantified using ImageJ analysis software.

### 4.7 Immunofluorescence staining

Mice were perfused transcardially with PBS (pH 7.4) followed by 4% PFA, and then the brain samples were fixed in 4% PFA for 24 h. Human hippocampal tissue was fixed in 4% PFA for 48 h. Immunofluorescence was performed in brain sections as previously described (Tao et al., 2021). The brain sections were incubated at 4°C overnight with the primary antibodies as follows: rabbit anti IBA‐1 (1:500, Abcam, ab178846); rabbit anti NEUN (1:500, Abcam, ab177487); chicken anti GFAP (1:500, Abcam, ab4674); mouse anti‐AIM2 (1:200, Santa Cruz Biotechnology, sc‐515514); rat anti‐CD68 (1:500, Abcam, ab53444); mouse anti‐CD68 (1:200, Bio Rad, MCA1957GA); mouse anti‐PSD‐95 (1:1000, Abcam, ab2723); chicken anti‐MAP‐2 (1:1000, Abcam, ab5392); rat anti‐C1q (1:500, Abcam, ab11861); rat anti‐C3 (1:500, Abcam, ab11862). The samples were washed with PBS three times (10 min each) and incubated with secondary antibodies (Invitrogen, 1:500) at RT for 2 h, followed by 20 min of DAPI staining (1:1000, Bioworld) for nuclear visualization. Fluorescent images were collected with a confocal microscope (Olympus, FV1200, Olympus Corporation) and analyzed using Image J software. For 3D image analyses, 3D reconstitution images were processed with software Imaris (Bitplane). For immunostaining of primary cultured cell, cells were fixed for 15 min with 4% PFA, and the remaining steps were the same as immunofluorescence staining of brain tissues.

### 4.8 Electrophysiology

Acute 300 μm hippocampal slices were prepared as described previously(Tao et al., 2021). The slices were incubated in artificial cerebrospinal fluid (ACSF) constantly bubbled with 95% O_2_ and 5% CO_2_ for at least 1 h at room temperature before recordings. For field recordings, the slices were placed into the microelectrode array and perfused continuously with oxygenated ACSF at a flow of 2 mL/min at 32°C. fEPSP recordings were conducted from the stratum radiatum region of DG with MEA‐2100‐60‐System (Multi Channel Systems). Input–output relationships of synapses were analyzed by measuring the slope of fEPSPs. A magnitude of 50% of the maximum evoked response was used as the stimulation intensity in LTP experiments. After 30 min of stable baseline, the LTP was induced with 100‐Hz high‐frequency stimulation (HFS; three trains, 1 s duration, 10 s interval time). The initial slopes of fEPSP were measured and normalized by the averaged slope value at baseline. The LTP‐Director software was used for data acquisition, and the data were analyzed using the LTP‐Analyzer software. In addition, to assess mEPSCs, we performed whole‐cell patch clamp experiments with borosilicate glass pipettes (resistance of 4–6 MΩ) filled with internal solution (125 mM potassium gluconate, 5 mM KCl, 1 mM MgCl_2_·6H_2_O, 0.2 mM EGTA, 10 mM HEPES, 10 mM Na2‐phosphocreatine, 4 mM Mg‐ATP and 0.3 mM Na‐GTP). Furthermore, mEPSCs were recorded in the presence of tetrodotoxin (1 μM) and bicuculline (20 μM) at a holding potential of −70 mV. The hippocampal DG neurons were visualized through IR‐DIC using an upright microscope with a 40× water‐immersion lens (Olympus) and Iris 9 Scientific CMOS (sCMOS) camera (Teledyne Photometrics) during recording, and the signals were recorded with MutiClamp, 700B amplifiers, Digidata 1550B analog‐to‐digital converters and pClamp 10.7 software (Molecular Devices). The mEPSCs events were detected by Clampfit 10.5 software (Molecular Devices), where the threshold for detection was set just above the baseline noise of the recordings, which was <5 pA, well below the average and median amplitude of the mEPSCs.

### 4.9 Golgi staining and sholl analysis

For Golgi staining, the brain tissues of mice were processed with a FD Rapid Golgi stain kit (FD Neurotechnologies) as previously described (Tao et al., 2021). Afterwards, the samples were coronally sliced (100 μm) using cryostat microtome (Leica) for staining according to manufacturer's protocol. The images were captured with Olympus IX73 and sholl and dendritic spine analyses were performed with ImageJ software (Fiji, NIH).

### 4.10 Flow cytometric analysis of microglia

The primary microglia transfected with lentivirus were cultured with 580/605 fluorescent beads diluted 1:1000 with media from Invitrogen (1 μm diameter) for 30 min at 37°C, protected from light. After removing the mixtures, the cells were washed three times with PBS and centrifuged at 751 *g* for 5 min at 4°C. The supernatant was discarded and PBS was added to resuspend the cells. Flow cytometry was conducted with BD Accuri C6 Flow Cytometer (BD Biosciences). Data were analyzed using FlowJo v 10 (Tree Star).

### 4.11 Statistical analysis

All results were expressed as means ± SEM and analysed using SPSS 17.0 (SPSS). Comparisons between two groups were performed using unpaired Student's *t* test, while comparisons between more than two groups were performed using one‐ or two‐way analysis of variance (ANOVA) with repeated measures followed by Bonferroni post hoc test. Statistical significance was determined at *p* < 0.05.

## AUTHOR CONTRIBUTIONS

Yun Xu, Feng Bai, Xiaolei Zhu, Lei Ye and Shu Shu initiated, designed the study and wrote the manuscript. Lei Ye and Shu Shu conducted behavioral experiments, electrophysiological experiments and data analysis. Lei Ye, Junqiu Jia, Min Sun, Siyi Xu and Meijuan Zhang performed molecular biology experiments. Xinyu Bao performed isolation and culture of primary microglia and primary neuron. Huijie Bian and Yi Liu performed the sample collection. All authors approved the manuscript prior to submission.

## FUNDING INFORMATION

This study was funded by the National Natural Science Foundation of China (82271480, 81901091), the National Science and Technology Innovation 2030—Major program of “Brain Science and Brain‐Like Research” (2022ZD0211800 to YX), the National Natural Science Foundation of China (82130036 and 81920108017 to YX), the Key Research and Development Program of Jiangsu Province of China (BE2020620 to YX), Jiangsu Province Key Medical Discipline (ZDXKA2016020 to YX).

## CONFLICT OF INTEREST STATEMENT

The authors declare that they have no conflict of interest.

## Supporting information


Figure S1‐S20

Appendix S1

## Data Availability

The data that support the findings of the current study are available from the corresponding author upon reasonable request.
