# Supplementary material for: Absent in melanoma 2 mediates aging‐related cognitive dysfunction by acting on complement‐dependent microglial phagocytosis
Source: Aging Cell. 2023 May 12;22(7):e13860. doi: 10.1111/acel.13860 (PMC10352562; doi:10.1111/acel.13860)
Supplement: Supplementary file 1 — Figure S1‐S20 [file ACEL-22-e13860-s001.pdf]

Figure S1

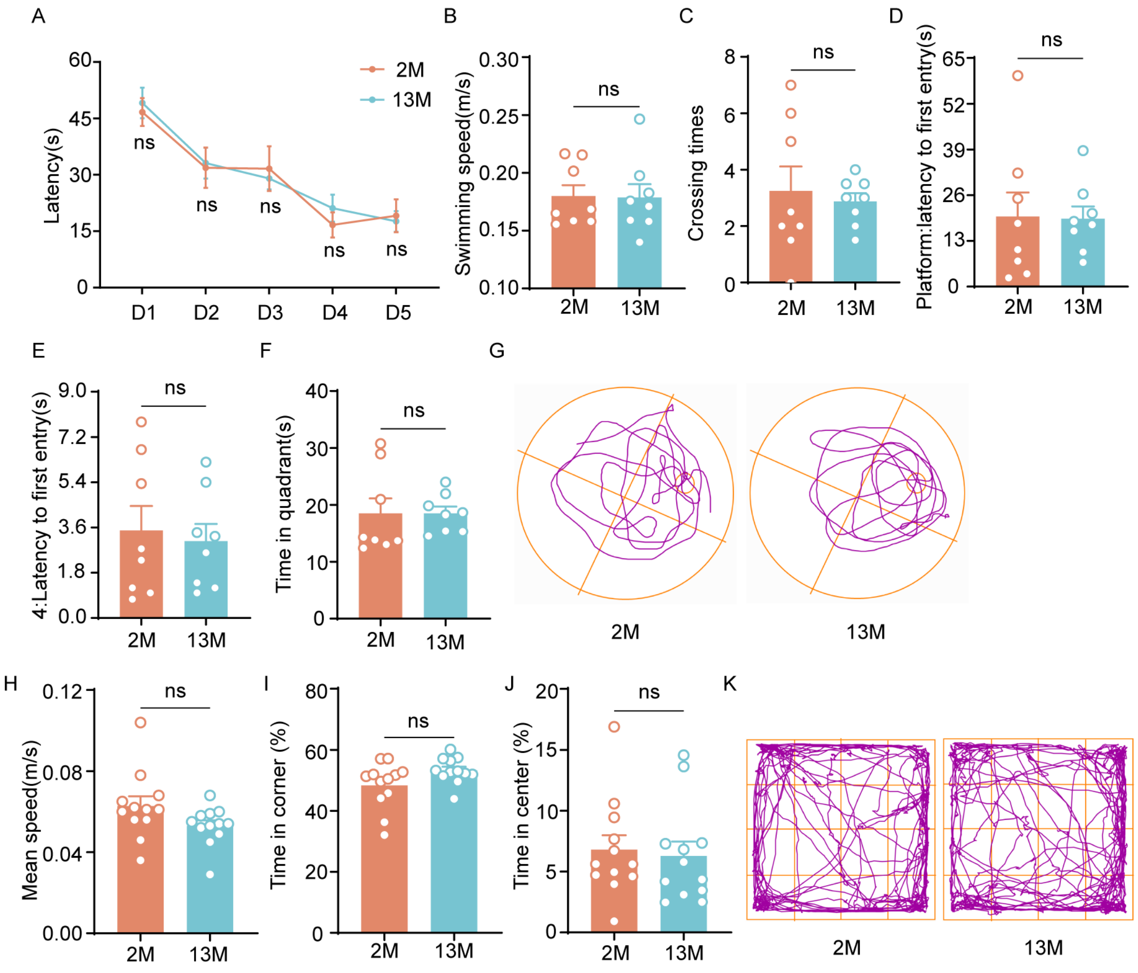
A
B
C
D
Latency(s)
2M
13M
D1
D2
D3
D4
D5
ns
Swimming speed(m/s)
Crossing times
Platform:latency to first entry(s)
E
F
G
4:Latency to first entry(s)
Time in quadrant(s)
H
I
J
K
Mean speed(m/s)
Time in corner (%)
Time in center (%)

Figure S2

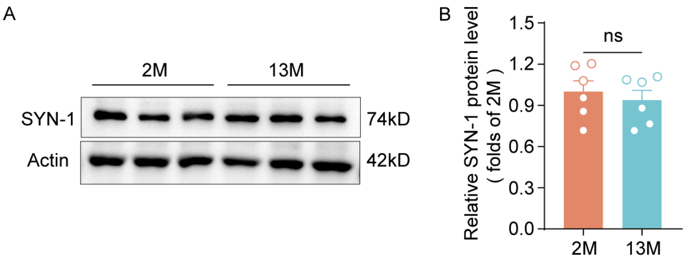

A
2M
13M
SYN-1
74kD
Actin
42kD
B
Relative SYN-1 protein level
(folds of 2M)
1.5
1.2
0.9
0.6
0.3
0.0
ns
2M
13M

Figure S3

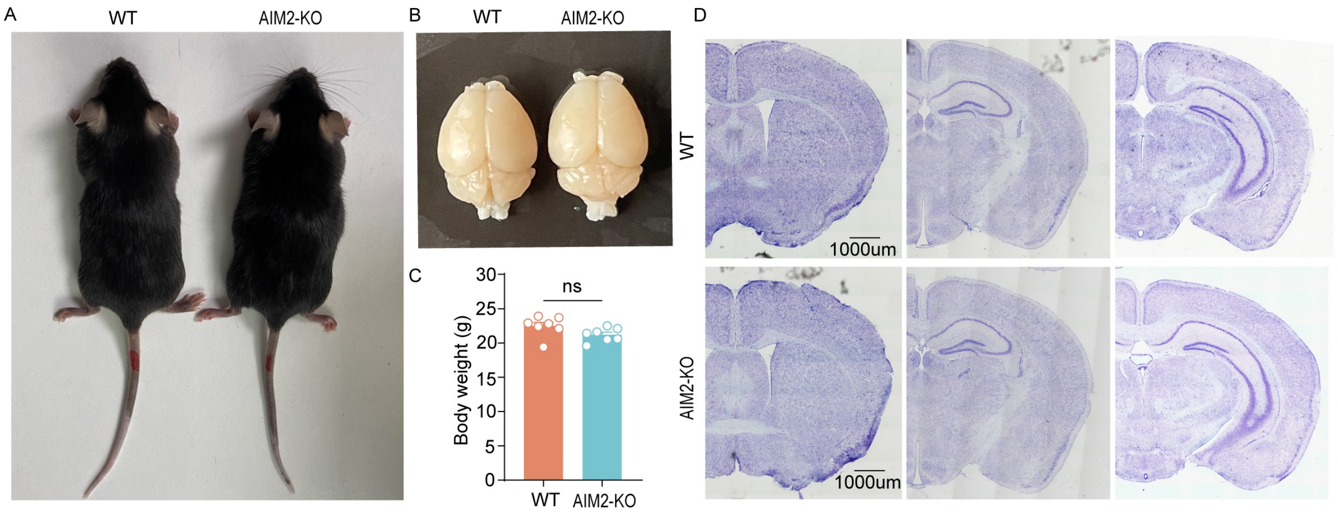
A
WT
AIM2-KO
B
WT
AIM2-KO
C
ns
Body weight (g)
30
25
20
15
10
5
0
WT
AIM2-KO
D
WT
AIM2-KO
1000um
1000um

Figure S4

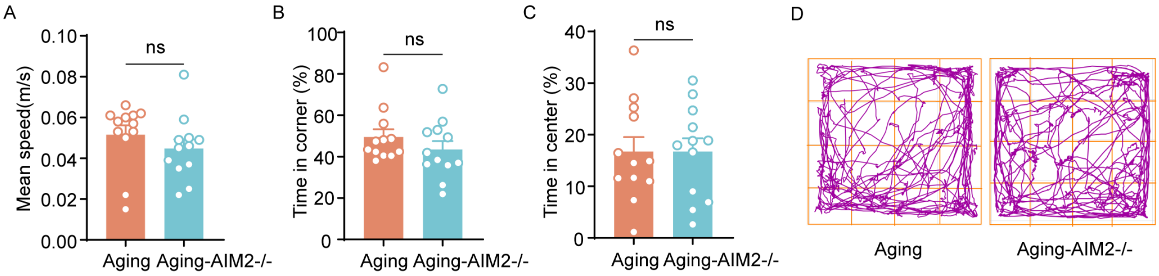

A
Mean speed(m/s)
0.10
0.08
0.06
0.04
0.02
0.00
ns
Aging
Aging-AIM2-/-
B
Time in corner (%)
100
80
60
40
20
0
ns
Aging
Aging-AIM2-/-
C
Time in center (%)
40
30
20
10
0
ns
Aging
Aging-AIM2-/-
D
Aging
Aging-AIM2-/-

Figure S5

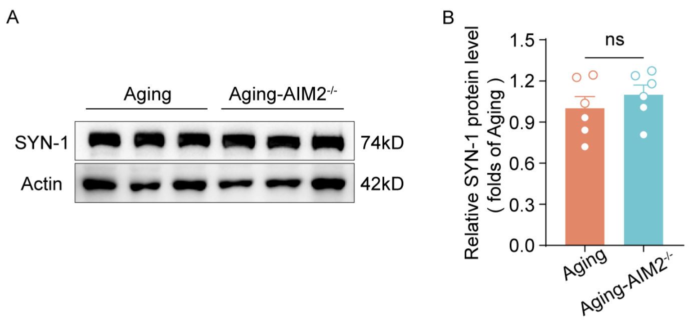

A
Aging
Aging-AIM2-/-
SYN-1
Actin
74kD
42kD
B
Relative SYN-1 protein level
(folds of Aging)
ns
1.5
1.2
0.9
0.6
0.3
0.0
Aging
Aging-AIM2-/-

Figure S6

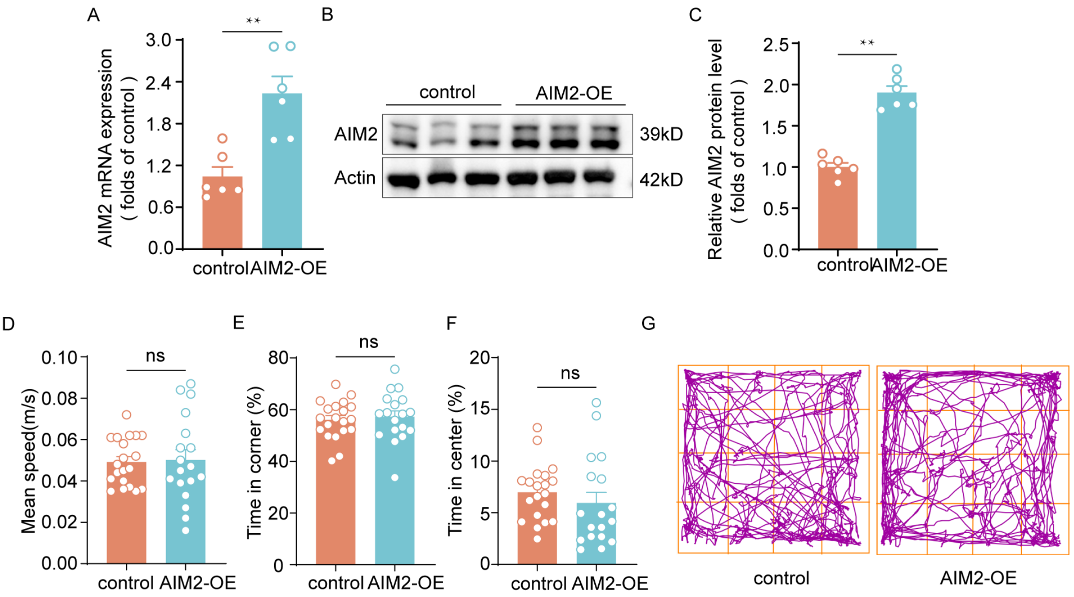

A
AIM2 mRNA expression (folds of control)
3.0
2.4
1.8
1.2
0.6
0.0
**
control
AIM2-OE
B
control
AIM2-OE
AIM2
Actin
39kD
42kD
C
Relative AIM2 protein level (folds of control)
2.5
2.0
1.5
1.0
0.5
0.0
**
control
AIM2-OE
D
Mean speed(m/s)
0.10
0.08
0.06
0.04
0.02
0.00
ns
control
AIM2-OE
E
Time in corner (%)
80
60
40
20
0
ns
control
AIM2-OE
F
Time in center (%)
20
15
10
5
0
ns
control
AIM2-OE
G
control
AIM2-OE

Figure S7

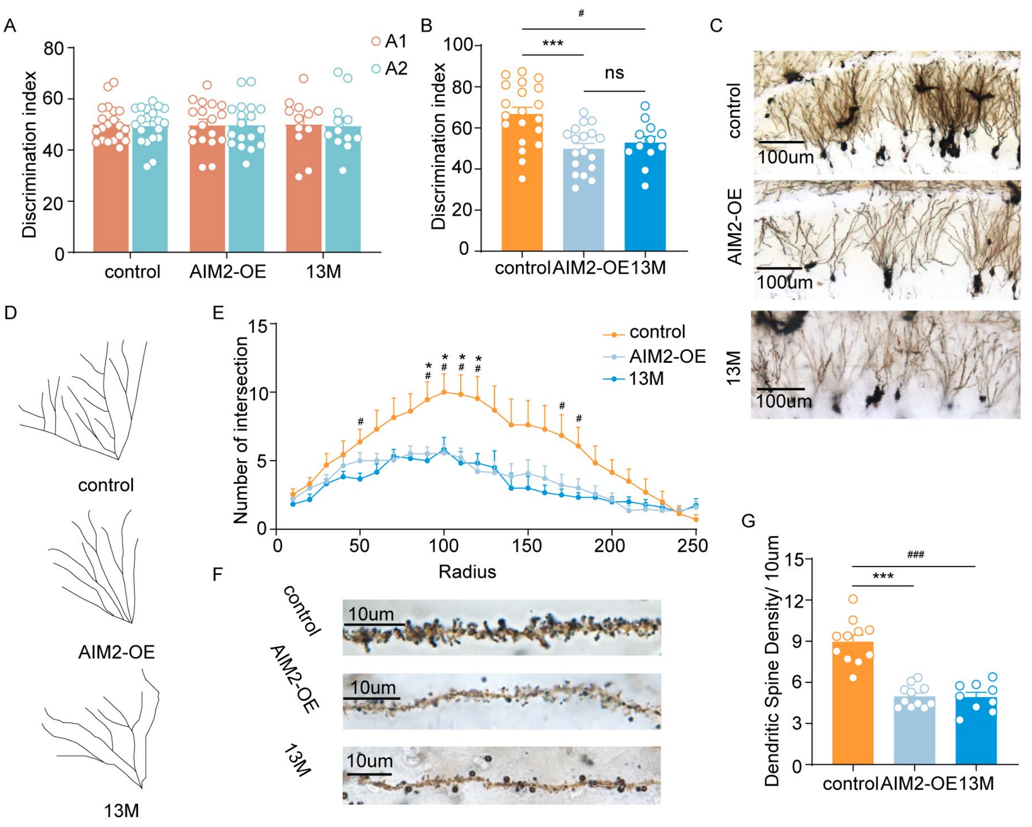
A
A1
A2
Discrimination index
0
20
40
60
80
control
AIM2-OE
13M
B
Discrimination index
0
20
40
60
80
100
***
#
ns
control
AIM2-OE
13M
C
control
AIM2-OE
13M
100um
D
control
AIM2-OE
13M
E
Number of intersection
Radius
control
AIM2-OE
13M
F
control
AIM2-OE
13M
10um
G
Dendritic Spine Density/ 10um
***
###
control
AIM2-OE
13M

Figure S8

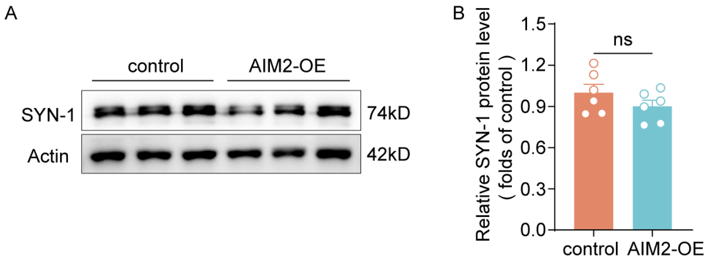

A
control
AIM2-OE
SYN-1
74kD
Actin
42kD
B
Relative SYN-1 protein level
(folds of control)
1.5
1.2
0.9
0.6
0.3
0.0
ns
control
AIM2-OE

Figure S9

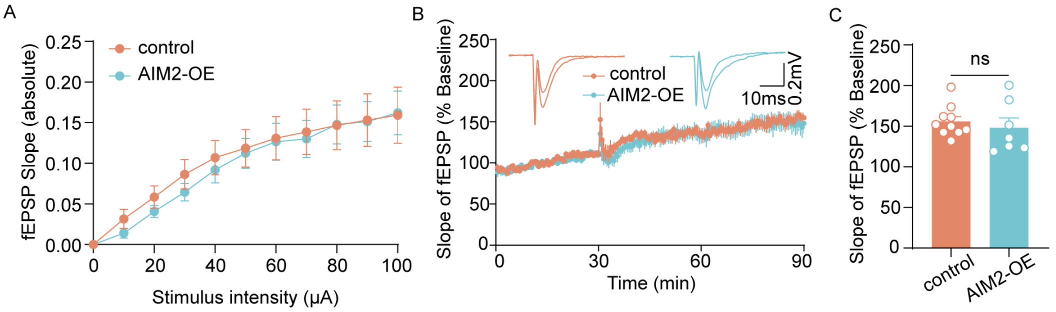

A
B
C
control
AIM2-OE
fEPSP Slope (absolute)
0.25
0.20
0.15
0.10
0.05
0.00
0
20
40
60
80
100
Stimulus intensity (μA)
Slope of fEPSP (% Baseline)
250
200
150
100
50
0
30
60
90
Time (min)
10ms
0.2mV
ns

Figure S10

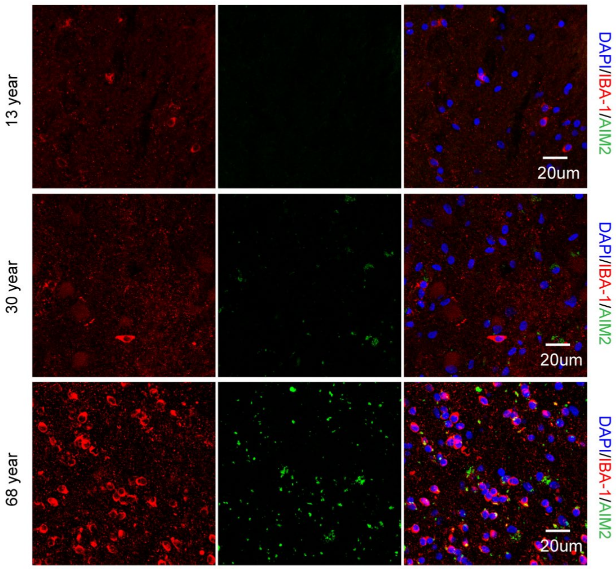

Figure S11

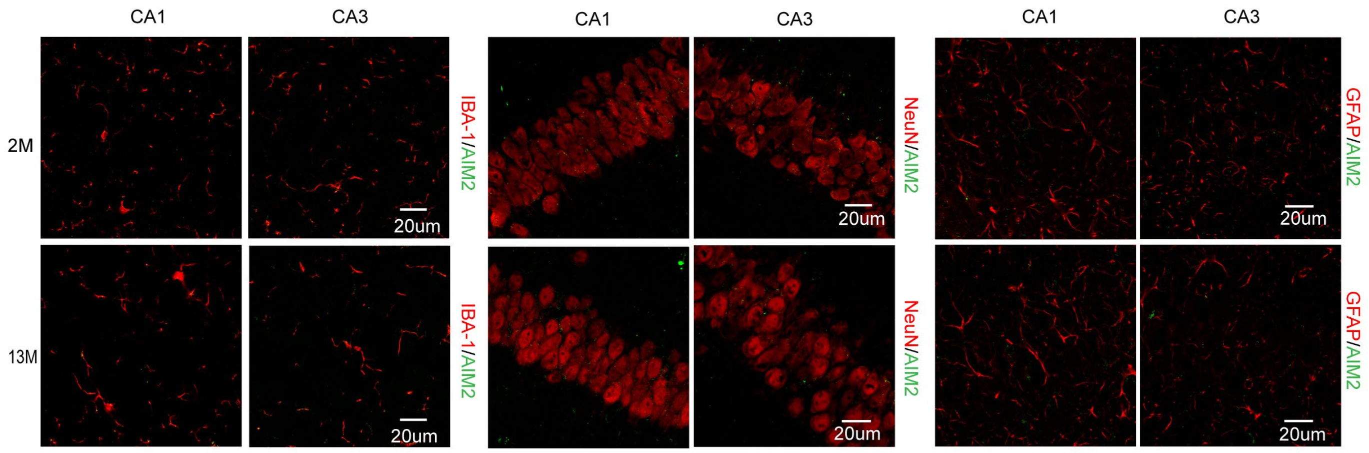

CA1
CA3
CA1
CA3
CA1
CA3
2M
13M
IBA-1/AIM2
NeuN/AIM2
GFAP/AIM2
20um

Figure S12

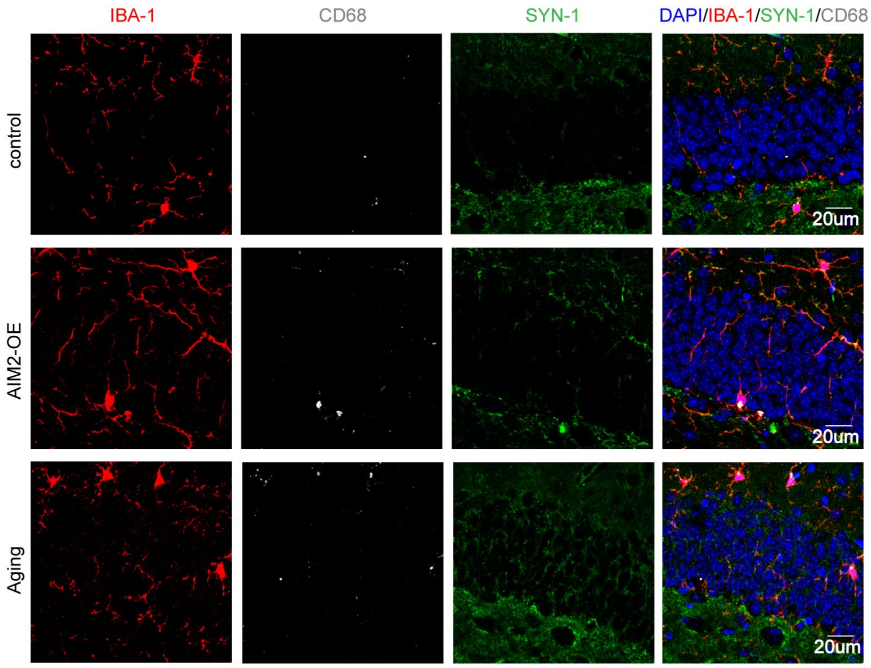
IBA-1
CD68
SYN-1
DAPI/IBA-1/SYN-1/CD68
control
AIM2-OE
Aging
20um
20um
20um

Figure S13

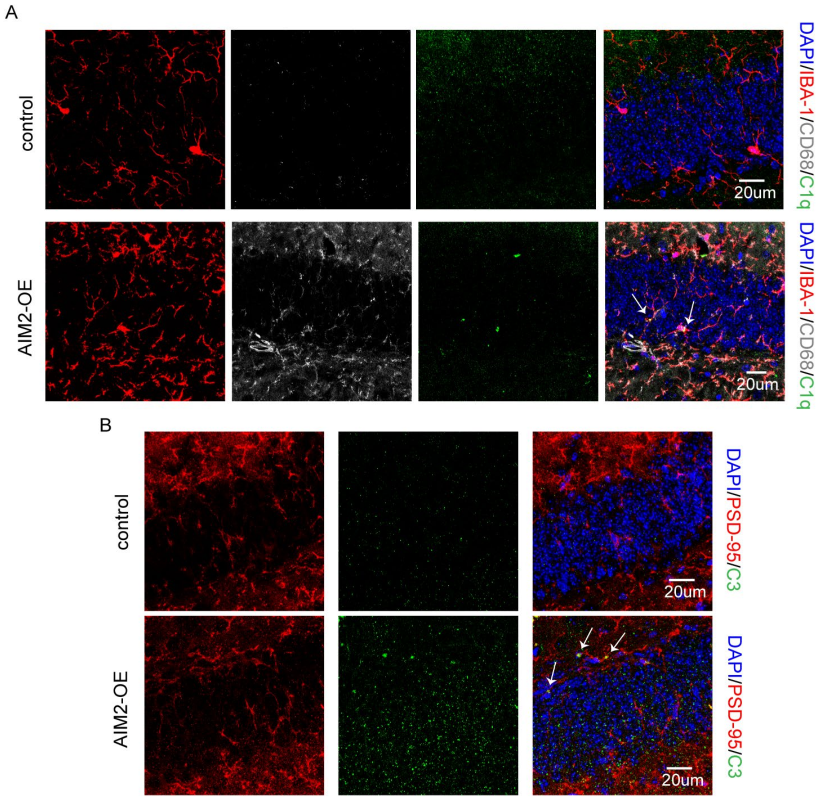

Figure S14

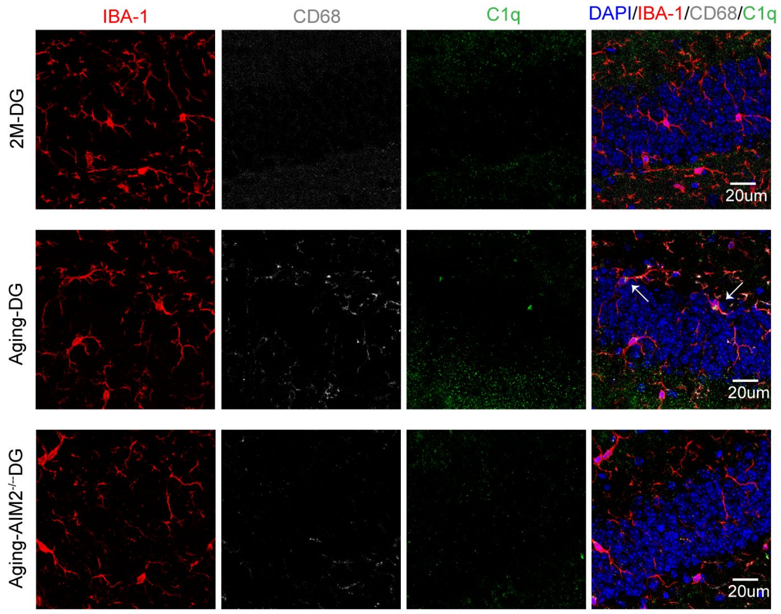

IBA-1
CD68
C1q
DAPI/IBA-1/CD68/C1q
2M-DG
Aging-DG
Aging-AIM2-/--DG
20um
20um
20um

Figure S15

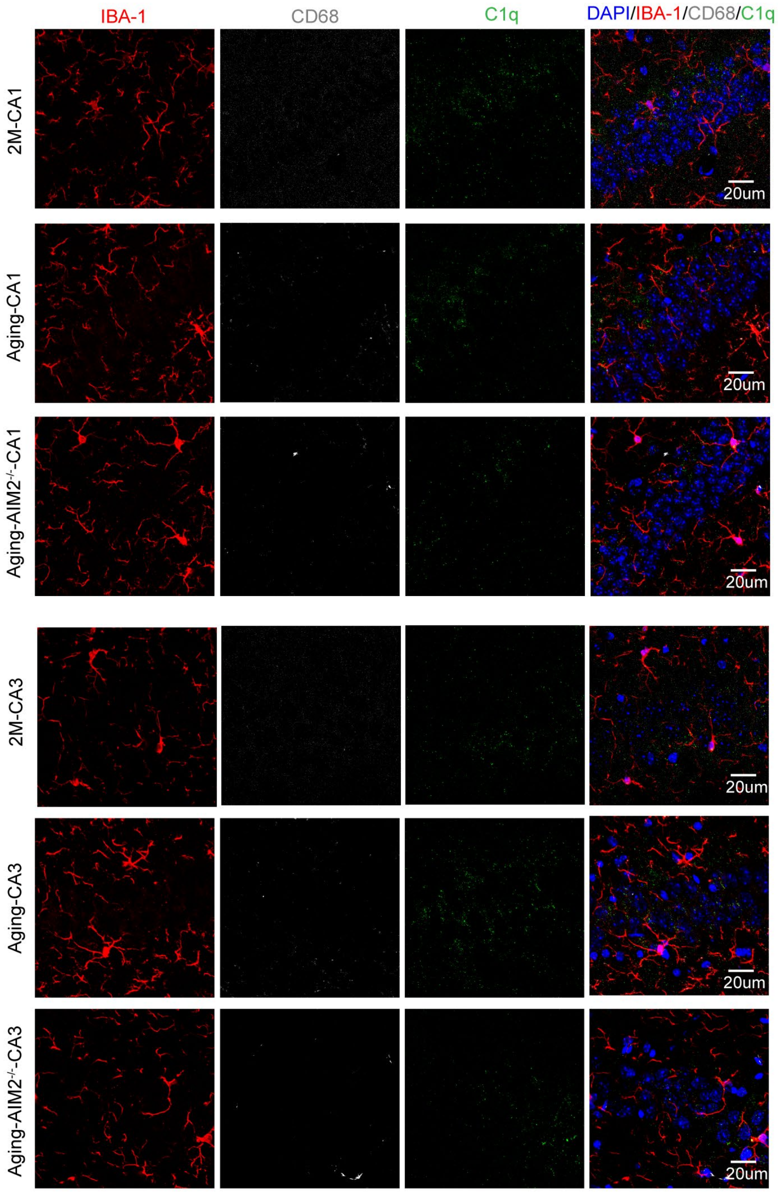
IBA-1
CD68
C1q
DAPI/IBA-1/CD68/C1q
2M-CA1
Aging-CA1
Aging-AIM2-/--CA1
2M-CA3
Aging-CA3
Aging-AIM2-/--CA3
20um

Figure S16

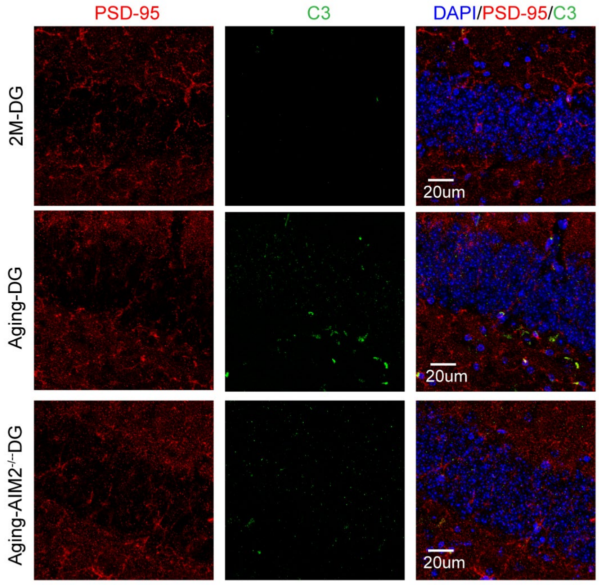

PSD-95
C3
DAPI/PSD-95/C3
2M-DG
Aging-DG
Aging-AIM2-/-DG
20um
20um
20um

Figure S17

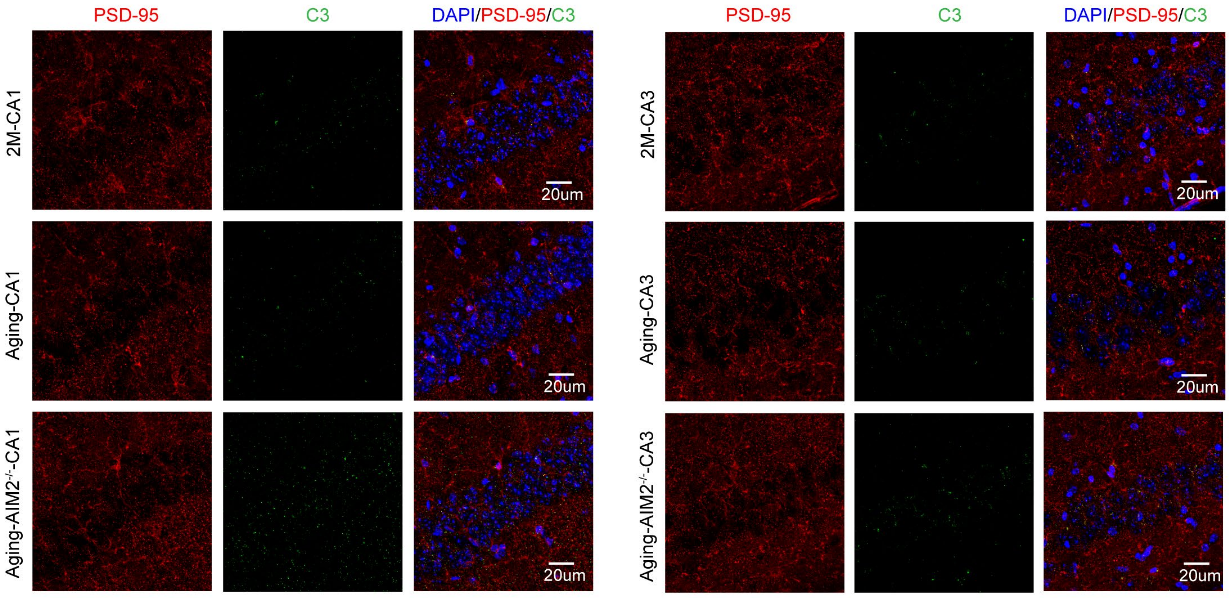

PSD-95
C3
DAPI/PSD-95/C3
PSD-95
C3
DAPI/PSD-95/C3
2M-CA1
Aging-CA1
Aging-AIM2-/--CA1
2M-CA3
Aging-CA3
Aging-AIM2-/--CA3
20um

Figure S18

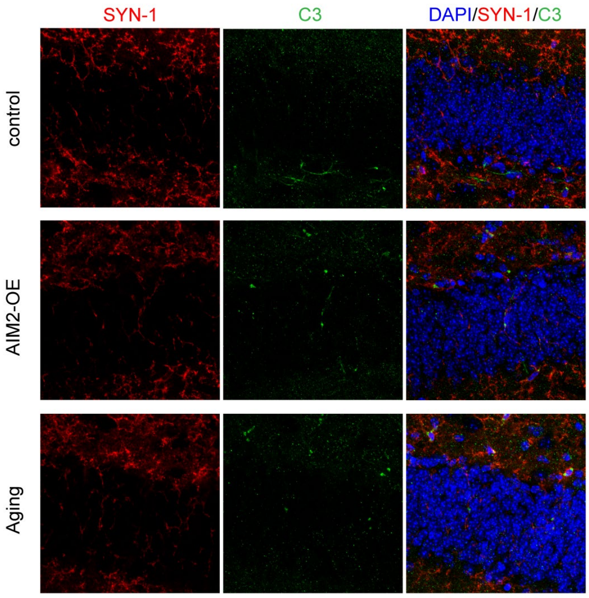

Figure S19

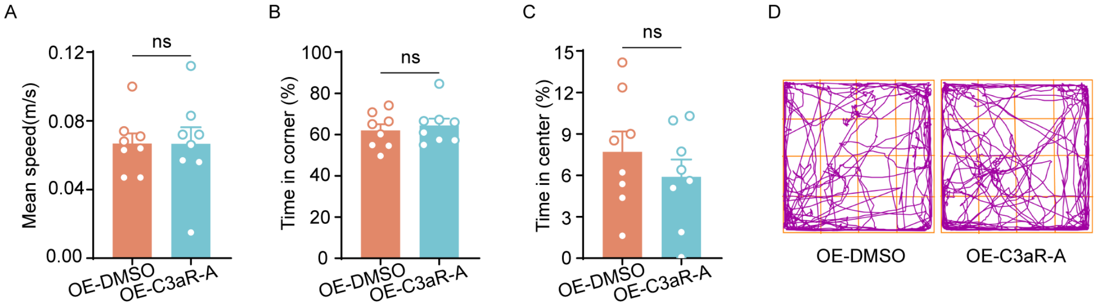
A
B
C
D
Mean speed(m/s)
0.12
0.08
0.04
0.00
ns
Time in corner (%)
100
80
60
40
20
0
Time in center (%)
15
12
9
6
3
OE-DMSO
OE-C3aR-A

Figure S20

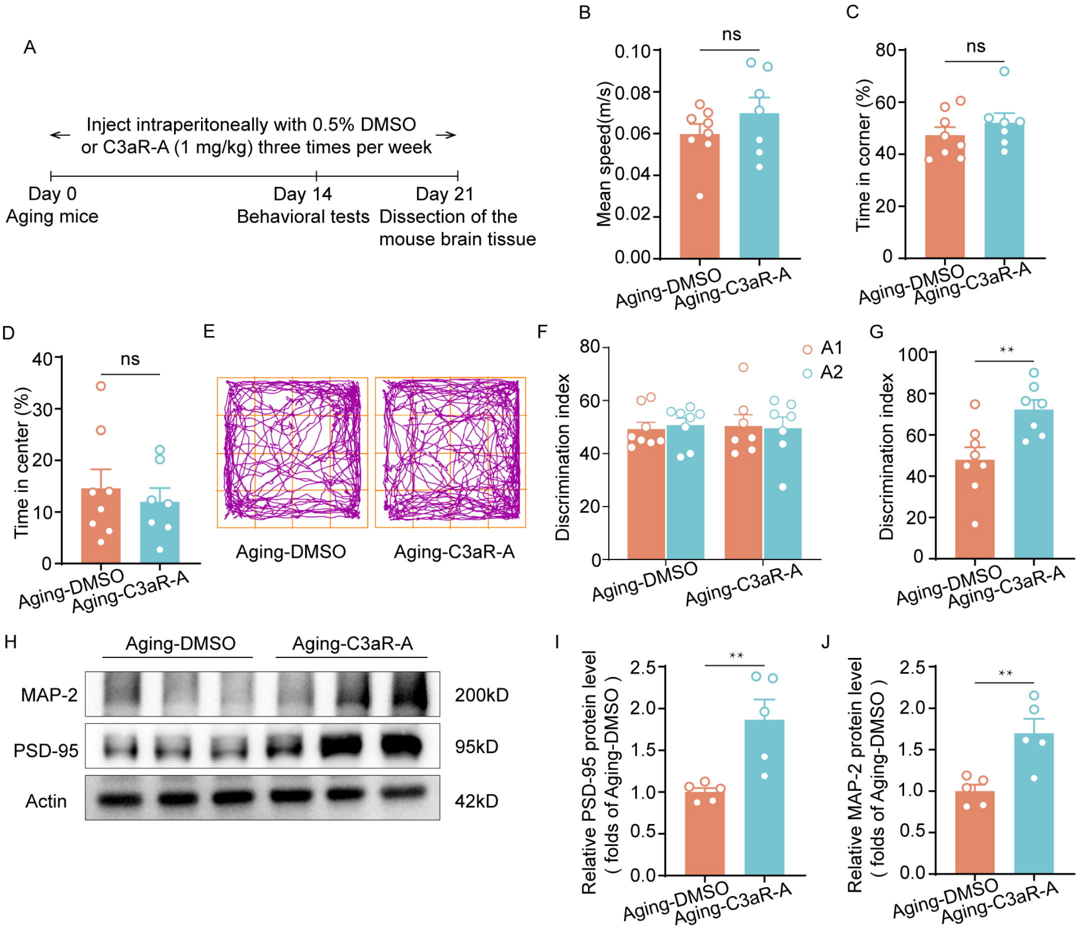

A
Inject intraperitoneally with 0.5% DMSO
or C3aR-A (1 mg/kg) three times per week
Day 0
Aging mice
Day 14
Behavioral tests
Day 21
Dissection of the
mouse brain tissue
B
ns
Mean speed(m/s)
0.10
0.08
0.06
0.04
0.02
0.00
Aging-DMSO
Aging-C3aR-A
C
ns
Time in corner (%)
80
60
40
20
0
Aging-DMSO
Aging-C3aR-A
D
ns
Time in center (%)
40
30
20
10
0
Aging-DMSO
Aging-C3aR-A
E
Aging-DMSO
Aging-C3aR-A
F
Discrimination index
80
60
40
20
0
A1
A2
Aging-DMSO
Aging-C3aR-A
G
**
Discrimination index
100
80
60
40
20
0
Aging-DMSO
Aging-C3aR-A
H
Aging-DMSO
Aging-C3aR-A
MAP-2
200kD
PSD-95
95kD
Actin
42kD
I
**
Relative PSD-95 protein level
(folds of Aging-DMSO)
2.5
2.0
1.5
1.0
0.5
0.0
Aging-DMSO
Aging-C3aR-A
J
**
Relative MAP-2 protein level
(folds of Aging-DMSO)
2.5
2.0
1.5
1.0
0.5
0.0
Aging-DMSO
Aging-C3aR-A
