# Supplementary material for: Absent in melanoma 2 mediates aging‐related cognitive dysfunction by acting on complement‐dependent microglial phagocytosis
Source: Aging Cell. 2023 May 12;22(7):e13860. doi: 10.1111/acel.13860 (PMC10352562; doi:10.1111/acel.13860)
Supplement: Supplementary file 2 — Appendix S1 [file ACEL-22-e13860-s002.docx]

**Supporting information**

AIM2 mediates aging-related cognitive dysfunction by acting complement-dependent microglial phagocytosis

Lei Ye, Shu Shu, Junqiu Jia, Min Sun, Siyi Xu, Xinyu Bao, Huijie Bian, Yi Liu, Meijuan Zhang, Xiaolei Zhu, Feng Bai, Yun Xu

**Supplemental Figure**

**Figure S1 Aging mice did not display significant differences in working memory in water maze, locomotor activity and anxiety-related behaviors.**

(A) The escape latency of 2-month-old and 13-month-old male mice was analyzed in the acquisition trial in the MWM tests. n = 8 for each group. F(1,14) = 0.03921, p = 0.8459**.** (B-F) The swimming speed (B), the number of platform crossings (C), latency to the target platform (D), latency to the target quadrant (E) and time in the target quadrant (F) of 2-month-old and 13-month-old male mice were recorded in the probe trial. n = 8 for each group. t(14) = 0.07589, p = 0.9406 for swimming speed; t(14) = 0.4099, p = 0.6881 for the number of platform crossings; t(14) = 0.07909, p = 0.9381 for latency to the target platform; t(14) = 0.3569, p = 0.7265 for latency to the target quadrant; t(14) = 0.000, p > 0.9999 for time in the target quadrant. (G) Typical images of tracing path in the probe test. (H-J) In the Open field test, mean speed (H) and time spent in the corner (I) and center (J) zone were recorded. n = 12 for each group. t(22) = 1.757, p = 0.0929 for mean speed; t(22) = 1.944, p = 0.0648 for time spent in the corner; t(22) = 0.3124, p = 0.7577 for time spent in the center. (K) Representative tracing path for each group in the Open field test. Data were shown as mean ± SEM. Unpaired two-tailed t-test for B-F and H-J. Two-way ANOVA followed by Bonferroni’s post hoc test for A. ns no significance.

**Figure S2 Aging mice did not exhibit a detectable change in SYN-1 expression from the DG region.**

(A) SYN-1 protein expression levels in the DG of 2-month-old and 13-month-old male mice were analyzed using immunoblot assays. (B) Quantification of the protein expression levels of SYN-1 normalized to β-actin as a loading control. n = 6 for each group. t(10) = 0.5918, p = 0.5672. Data were shown as mean ± SEM. Unpaired two-tailed t-test for B. ns no significance.

**Figure S3 AIM2-KO mice displayed normal body weight and gross brain structure.**

(A-B) Representative images showing overall appearance and brain of AIM2-KO and WT mice. (C) Quantification of body weight for AIM2-KO and WT mice. n = 7 for each group. t (12) = 0.8150, p = 0.4310. (D) Representative Nissl-staining images of brains from AIM2-KO and WT mice. Data were shown as mean ± SEM. Unpaired two-tailed t-test for C, ns no significance.

**Figure S4 The knockout of AIM2 in aging mice had no impact on motor function and anxiety levels.**

(A-D) In the Open field test, mean speed (A) and time spent in the corner (B) and center (C) zone of 13-month-old WT and AIM2^-/-^ male mice were recorded. n = 12 for each group. t(22) = 1.043, p = 0.3085 for mean speed; t(22) = 1.100, p = 0.2830 for time spent in the corner; t(22) = 0.001363, p = 0.9989 for time spent in the center. (D) Representative tracing path for each group in the Open field test. Data were shown as mean ± SEM. Unpaired two-tailed t-test for A-C. ns no significance.

**Figure S5 AIM2^-/-^ mice did not exhibit a detectable change in SYN-1 expression from the DG region.**

(A) SYN-1 protein expression levels in the DG of 13-month-old WT and AIM2^-/-^ male mice were analyzed using immunoblot assays. (B) Quantification of the protein expression levels of SYN-1 normalized to β-actin as a loading control. n = 6 for each group. t(10) = 0.8878, p = 0.3955. Data were shown as mean ± SEM. Unpaired two-tailed t-test for B. ns no significance.

**Figure S6 AIM2 overexpression in vivo did not affect motor activity and anxiety performance.**

(A-C) The overexpression efficiency of the lentivirus was determined through detecting the AIM2 level in DG region via quantitative real-time PCR (A) and western blotting (B and C). n = 6 for each group. t(10) = 4.247, p = 0.0017 for QPCR. t(10) = 9.511, p < 0.0001 for western blotting. (D-F) In the Open field test, mean speed (D) and time spent in the corner (E) and center (F) zone were recorded. n = 18-20 for each group. t (36) =0.1788, p = 0.8591 for mean speed; t(36) = 0.4447, p = 0.6592 for time spent in the corner; t(36) = 0.8887, p = 0.3801 for time spent in the center. (G) Representative tracing path for each group in the Open field test. Data were shown as mean ± SEM. Unpaired two-tailed t-test for A, C and D-F. **p < 0.01; ns no significance.

**Figure S7** **Overexpression of AIM2 in the DG of 2-month-old mice brings similar changes as that of 13-month-old mice.**

(A and B) The time spent exploring two identical objects during the training phase (A) and the displaced object during the test session (B) in the OPS test of control mice, AIM2-OE mice and 13-month-old male mice were recorded. n = 12-20 for each group. F (2, 47) = 1.563, AIM2-OE group vs. control group: p = 0.0005, 13-month-old group vs. control group: p = 0.0112. (C) Overview of the DG region in control mice, AIM2-OE mice and 13-month-old male mice. (D) Neuronal tracing in control mice, AIM2-OE mice and 13-month-old male mice. (E) Quantification of the numbers of neuronal intersections in control (n = 2-3 neurons / mouse, N = 5 mice / group), AIM2-OE (n = 2-3 neurons / mouse, N = 5 mice / group) and 13-month-old (n = 2-3 neurons / mouse, N = 3 mice / group) male mice by Sholl analysis. F (2, 30) = 3.310, AIM2-OE group vs. control group: p < 0.0001, 13-month-old group vs. control group: p < 0.0001. (F) Representative images of dendrite morphology. (G) Quantitative analysis of mean spine density in control (n = 2-3 spines / mouse, N = 5 mice / group), AIM2-OE (n = 2-3 spines / mouse, N = 5 mice / group) and 13-month-old (n = 3 spines / mouse, N = 3 mice / group) male mice. F (2, 28) = 1.747, AIM2-OE group vs. control group: p < 0.0001, 13-month-old group vs. control group: p < 0.0001. Data were shown as mean ± SEM. One-way ANOVA followed by Dunnett’s post hoc test for B and G. Two-way ANOVA followed by Bonferroni’s post hoc test for E. *p < 0.05, ***p < 0.001 for AIM2-OE group vs. control group; #p < 0.05, ###p < 0.001 for 13-month-old group vs. control group.

**Figure S8 AIM2 overexpression in vivo did not affect SYN-1 expression from the DG region.**

(A) SYN-1 protein expression levels in the DG of control and AIM2-OE mice were analyzed using immunoblot assays. (B) Quantification of the protein expression levels of SYN-1 normalized to β-actin as a loading control. n = 6 for each group. t(10) = 1.310, p = 0.2194. Data were shown as mean ± SEM. Unpaired two-tailed t-test for B. ns no significance.

**Figure S9 AIM2 overexpression in vivo did not affect basal synaptic transmission or synaptic plasticity in the CA1 region.**

(A) The I/O curve for AIM2-OE mice (n = 2-3 slices / mouse, N = 3 mice / group) did not differ significantly from the control mice (n = 1-3 slices / mouse, N = 4 mice / group). F (1, 13) = 0.03796, P = 0.8485. (B and C) LTP of hippocampal slices was evaluated in the CA1 region of control (n = 2-3 slices / mouse, N = 4 mice / group) and AIM2-OE (n = 2-3 slices / mouse, N = 3 mice / group) mice. The slope of the regression line was not significantly altered after overexpression of AIM2. t(15) = 0.6237, p = 0.5422. Data were shown as mean ± SEM. Two-way ANOVA followed by Bonferroni’s post hoc test for A. Unpaired two-tailed t-test for C. ns no significance.

**Figure S10 The levels of AIM2 in the microglia of young individuals and older individuals were examined by immunostaining.**

High levels of AIM2 (green) immunoreactivity in IBA-1+ (red) microglia were detected in the hippocampus of older individuals compared to young individuals.

**Figure S11 The expression of AIM2 in different cell types from the CA1 and CA3 regions of the hippocampus in 2-month-old and 13-month-old male mice.**

Double immunofluorescence staining of AIM2 (green) with IBA-1 (red), NeuN (red) and GFAP (red) in the CA1 and CA3 region of 2-month-old and 13-month-old male mice.

**Figure S12**

Immunostaining for IBA-1 (red), CD68 (gray), SYN-1 (green) in the DG region of control, AIM2-OE and 13-month-old male mice.

**Figure S13 The localization of C1q and C3 in the DG region of the hippocampus.**

Colocalization of C1q (green) with IBA-1 (red) and CD68 (gray), C3 (green) with PSD-95 (red) in control and AIM2-OE mice.

**Figure S14 The localization of C1q in the DG region of the hippocampus.**

Colocalization of C1q (green) with IBA-1 (red) and CD68 (gray) in the 2-month-old, 13-month-old WT and 13-month-old AIM2^-/-^ group.

**Figure S15 The localization of C1q in the CA1 and CA3 regions of the hippocampus.**

Colocalization of C1q (green) with IBA-1 (red) and CD68 (gray) in the 2-month-old, 13-month-old WT and 13-month-old AIM2^-/-^ group.

**Figure S16 The localization of C3 in the DG region of the hippocampus.**

Colocalization of C3 (green) with PSD-95 (red) in the 2-month-old, 13-month-old WT and 13-month-old AIM2^-/-^ group.

**Figure S17 The localization of C3 in the CA1 and CA3 regions of the hippocampus.**

Colocalization of C3 (green) with PSD-95 (red) in the 2-month-old, 13-month-old WT and 13-month-old AIM2^-/-^ group.

**Figure S18**

Colocalization of C3 (green) with SYN-1 (red) in the hippocampal DG of control, AIM2-OE mice and 13-month-old male mice.

**Figure S19 C3aR-A treatment did not affect the general locomotor activity and anxiety levels of AIM2-OE mice.**

(A-D) In the Open field test, mean speed (A) and time spent in the corner (B) and center (C) zone of OE-DMSO and OE-C3aR-A mice were recorded. n = 7 for each group. t(14) = 0.01100, p = 0.9914 for mean speed; t(14) = 0.5386, p = 0.5986 for time spent in the corner; t(14) = 0.9365, p = 0.3649 for time spent in the center. (D) Representative tracing path for each group in the Open field test. Data were shown as mean ± SEM. Unpaired two-tailed t-test for A-C. ns no significance.

**Figure S20 C3aR antagonist improved cognitive deficits and rescues synapse density in aging mice.**

(A) Flow chart of the experimental design. (B-E) In the Open field test, mean speed (B) and time spent in the corner (C) and center (D) zone of 13-month-old male mice injected with DMSO or C3aR-A were recorded. n = 8 for each group. t(13) = 1.149, p = 0.2713 for mean speed; t(13) = 0.9920, p = 0.3393 for time spent in the corner; t(13) = 0.5611, p = 0.5842 for time spent in the center. (E) Representative tracing path for each group in the Open field test. (F and G) The percentage of time exploring identical objects during the training phase (F) and displaced object during the test session (G) of 13-month-old male mice injected with DMSO or C3aR-A was recorded. n = 8 for each group. t(13) = 3.086, p = 0.0087. (H) Representative western blotting analysis of PSD-95 and MAP-2 expression in 13-month-old male mice injected with DMSO or C3aR-A mice. (I and J) Quantification of PSD-95 (I) and MAP-2 (J) protein level (relative to the β-actin level). n = 5 for each group. t(8) = 3.498, p = 0.0081 for PSD-95; t(8) = 3.682, p = 0.0062 for MAP-2. Data were shown as mean ± SEM. Unpaired two-tailed t-test for B-D, G, I and J. **p < 0.01; ns no significance.
